# Towards understanding the crystallization of photosystem II: influence of poly(ethylene glycol) of various molecular sizes on the micelle formation of alkyl maltosides

**DOI:** 10.1007/s11120-024-01079-5

**Published:** 2024-03-15

**Authors:** Frank Müh, Adrian Bothe, Athina Zouni

**Affiliations:** 1 Institut für Theoretische Physik, Johannes Kepler Universität Linz, Altenberger Strasse 69, 4040 Linz, Austria; 2 Institut für Molekularbiologie und Biophysik, ETH Zürich, HPK, Otto-Stern-Weg 5, CH-8093 Zurich, Switzerland; 3 Institut für Biologie, Humboldt Universität zu Berlin, Leonor-Michaelis-Haus, Philippstrasse 13, 10095 Berlin, Germany

**Keywords:** Critical micelle concentration, Detergent, Free energy, Photosystem II, Polyethylene glycol, Protein–detergent complex

## Abstract

The influence of poly(ethylene glycol) (PEG) polymers H–(O–CH_2_–CH_2_)_*p*_–OH with different average molecular sizes $p$ on the micelle formation of *n*-alkyl-β-D-maltoside detergents with the number of carbon atoms in the alkyl chain ranging from $10$ to $12$ is investigated with the aim to learn more about the detergent behavior under conditions suitable for the crystallization of the photosynthetic pigment–protein complex photosystem II. PEG is shown to increase the critical micelle concentration (CMC) of all three detergents in the crystallization buffer in a way that the free energy of micelle formation increases linearly with the concentration of oxyethylene units (O–CH_2_–CH_2_) irrespective of the actual molecular weight of the polymer. The CMC shift is modeled by assuming for simplicity that it is dominated by the interaction between PEG and detergent monomers and is interpreted in terms of an increase of the transfer free energy of a methylene group of the alkyl chain by 0.2 kJ mol^−1^ per 1 mol L^−1^ increase of the concentration of oxyethylene units at 298 K. Implications of this effect for the solubilization and crystallization of protein–detergent complexes as well as detergent extraction from crystals are discussed.

**Supplementary Information:**

The online version contains supplementary material available at 10.1007/s11120-024-01079-5.

## Introduction

Photosynthesis research rests to a large part on the determination of protein structures. Important complexes like photosystem II (PSII) are membrane proteins that need to be removed from the native membrane by using detergents that are able to cover the protein´s hydrophobic, originally membrane-facing surfaces to make them hydrophilic and the formed protein–detergent complex (PDC) water soluble (Golub et al. 2020, 2021, 2022). This solubilization is not only important for many sprectroscopic techniques to characterize light harvesting and charge separation as well as electron and proton transfer processes, but is also central to the structure determination based on X-ray crystallography (Michel 1983; Ostermeier and Michel 1997; Privé 2007) and cryo-electron microscopy (Thonghin et al. 2018).

PSII is a multi-subunit pigment–protein complex (Müh and Zouni 2020) situated in the thylakoid membrane of oxygenic photosynthetic organisms like cyanobacteria, algae, and higher plants (Nelson and Junge 2015; Junge 2019; Blankenship 2021). The importance of PSII derives from the fact that it harbors the water-oxidizing complex (WOC), a protein-ligated Mn_4_CaO_5_ cluster that catalyzes the removal of electrons and protons from water with oxygen being released into the atmosphere as a byproduct (Müh and Zouni 2011; Yano and Yachandra 2014; Shevela et al. 2023). The power required to oxidize water is taken from light, whose energy is delivered by light-harvesting complexes to the reaction center, where charge separation takes place (Cardona et al. 2012; Müh and Zouni 2020; Shevela et al. 2023). The excess electrons produced in the latter process are used to reduce plastoquinone (Cardona et al. 2012; Müh et al. 2012), while the protons originating from water contribute to the buildup of a proton motive force across the thylakoid membrane (Blankenship 2021). Much effort has been put in recent years into the elucidation of the mechanism of water oxidation in the WOC (Ibrahim et al. 2015, 2020; Kern et al. 2018, 2019; Hussein et al. 2021; Bhowmick et al. 2023). Here, X-ray crystallography plays a key role, as it can be performed at ambient temperatures and with a time resolution (Neutze et al. 2015; Kern et al. 2019; Orville 2020; de Wijn et al. 2022).

Like any membrane protein, PSII has to be extracted from the membrane with the help of detergents (Kermani 2021) for further purification. This process, yielding a stable dispersed membrane protein in an aqueous solution, is often referred to as solubilization. Lipids originating from the membrane are still present at this stage, but are largely removed in subsequent purification steps. In the resulting pure membrane protein solution, the detergent has to be present at a minimum concentration to ensure that the membrane protein remains monodisperse, i.e., does not form aggregates, which is a prerequisite for crystallization. Keeping the membrane protein in this way in solution in an aqueous environment is also sometimes called solubilization with the meaning of solubilizing protein aggregates. In the present work, we shall use the term “solubilization” in this latter sense. The minimal total detergent concentration required to keep the purified membrane protein in monodisperse solution is called the critical solubilization concentration (CSC) in this context (Müh and Zouni 2005).

In order to crystallize a protein like PSII, a precipitating agent like poly(ethylene glycol) (PEG) is added to modify the solubility of the protein and to force it into the formation of a crystal (Loll et al. 2005). As mentioned above, in the case of membrane proteins, detergents have to be present in order to keep the protein solubilized in the first place. However, detergents, as they are amphiphilic molecules, show a peculiar behavior in aqueous solution. Particularly, a certain minimal concentration, called the critical micelle concentration (CMC), needs to be exceeded to make the detergent work as solubilizing agent. Above the CMC, the detergent molecules aggregate into micelles (Wennerström and Lindman 1979; Nagarajan and Ruckenstein 1991; Chakraborty and Ghosh 2008; Dey et al. 2021) and also start forming belts surrounding the hydrophobic protein parts (Müh and Zouni 2008). It is hypothesized that the CSC, at which the detergent belts in all PDCs are supposed to be complete, is larger than the CMC depending on the number of detergent molecules per protein bound in the PDC (Müh and Zouni 2005, 2008; Müh et al. 2015). The CMC (and likely also the CSC) is very sensitive to co-solutes in the buffer (Aoudia and Zana 1998; Hitscherich et al. 2000; Blouwolff and Fraden 2007; Santonicola et al. 2007; Müh and Zouni 2008) and should be carefully characterized to optimize crystallization setups.

*n*-Alkyl-β-D-maltosides are an important class of mild, nonionic, protein-friendly detergents widely used in crystallographic studies of PSII (Loll et al. 2005; Broser et al. 2010; Umena et al. 2011) and other photosynthetic (Jordan et al. 2001; Stroebel et al. 2003; Mazor et al. 2015) as well as nonphotosynthetic membrane proteins (Palsdottir et al. 2003; Yernool et al. 2004; Murata et al. 2005; Murakami et al. 2006; Rodrigues et al. 2006; Ago et al. 2007; Molina et al. 2007). They belong to the group of sugar-based surfactants (Stubenrauch 2001) with the hydrophilic head being a maltose and the hydrophobic tail an *n*-alkyl chain connected by an ether linkage (Rosevear et al. 1980). The CMCs and aggregation numbers (average number of detergent molecules per micelle) of detergents from this class with different alkyl chain lengths have been determined under diverse conditions by employing various techniques (De Grip and Bovee-Geurts 1979; Drummond et al. 1985; Alpes et al. 1986; Warr et al. 1986; Cecutti et al. 1991; Tummino and Gafni 1993; Zhang et al. 1996; Dupuy et al. 1997; Aoudia and Zana 1998; Aveyard et al. 1998; Liljekvist and Kronberg 2000; Blouwolff and Fraden 2007; Lipfert et al. 2007; Kunji et al. 2008; Tsamaloukas et al. 2009; Jumpertz et al. 2011; Oliver et al. 2013). In these studies, the CMC is defined operationally by a breaking point in the experimental titration curve. It has been suggested that this may lead to uncertainties in the determined CMC values as the breaking points in the different methods may not correspond to exactly the same total detergent concentration (Al-Soufi et al. 2012). Recently, we introduced a refined definition of the CMC (Bothe et al. 2023) based on the third derivative of the function describing the dependence of the detergent monomer concentration on the total detergent concentration, motivated by earlier suggestions (Phillips 1955; Al-Soufi et al. 2012). We also showed that the breaking points of the experimental and theoretical curves coincide, if the CMC is determined by a fluorescence technique, in which a dye´s emission is increased upon entering the micelles once formed (De Vendittis et al. 1981; Abuin et al. 1997). The question remained open of how to connect the new theoretical approach to the analysis of the effect of PEG on the CMC.

In the framework of our long-term research aiming at a better understanding of the physical chemistry underlying the crystallization process (Müh and Zouni 2005, 2008; Müh et al. 2015; Bothe et al. 2023), we investigated the influence of PEG polymers of various molecular sizes on the CMC of alkyl maltosides. In one of the earlier studies, we analyzed only a subset of the data concerning PEG2000 (Müh et al. 2015). In the present work, we finally analyze the remaining data concerning the other PEG types—including a characterization of the molarity of highly concentrated PEG solutions contingent on the polymer molecular weight—and establish a connection to the refined definition of the CMC. We also discuss possible implications of the results for the CSC and membrane protein crystallization.

## Theory

### Thermodynamics of micelle formation

We start by defining the Gibbs free energy $G$ of the solution:

1 $$G={G}_{{\text{f}}}+{G}_{{\text{mix}}}+{G}_{{\text{int}}}$$

Here, ${G}_{{\text{f}}}$ is the free energy of formation given by

2 $${G}_{{\text{f}}}={N}_{{\text{W}}}{\mu }_{{\text{W}}}^{0}+{N}_{1}{\mu }_{1}^{0}+\sum_{\nu >1}{N}_{\nu }\nu {\mu }_{\nu }^{0}+{N}_{{\text{P}}}{\mu }_{{\text{P}}}^{0}+\sum_{\alpha }{N}_{\mathrm{\alpha }}{\mu }_{\mathrm{\alpha }}^{0}$$

where ${\mu }_{{\text{i}}}^{0}$ and ${N}_{{\text{i}}}$ are the standard chemical potential and particle number, respectively, of species ${\text{i}}$. As explained by Bothe et al. (2023), the standard chemical potential represents a Raoult´s law standard state for the solvent (water, ${\text{i}}={\text{W}}$) and a Henry´s law standard state for all the solutes. The index “1” stands for a detergent monomer and the index “$\nu$” for a micelle with $\nu$ detergent molecules. Note that ${\mu }_{\nu }^{0}$ is the standard chemical potential of one detergent molecule in the micelle and contains contributions from interactions between the detergent molecules in the micelle for $\nu >1$. The index “P” stands for PEG, and it is assumed that there is only one type of PEG molecule present at a time. Finally, the index “$\alpha$” counts all the other co-solutes that we do not consider explicitly such as buffer and salt. For the following, it is useful to introduce the total number of co-solutes (excluding PEG)

3 $${N}_{{\text{cos}}}=\sum_{\alpha }{N}_{\mathrm{\alpha }}$$

and the total number of detergent molecules

4 $${N}_{{\text{det}}}={N}_{1}+\sum_{\nu >1}{N}_{\nu }\nu$$

Note that ${N}_{\nu }$ is the number of micelles of size $\nu$. Then, the total particle number is

5 $${N}_{{\text{tot}}}={N}_{{\text{W}}}+{N}_{{\text{det}}}+{N}_{{\text{P}}}+{N}_{{\text{cos}}}$$

and we can define the mole fractions for all species except detergent as

6 $${X}_{{\text{i}}}={N}_{i}/{N}_{{\text{tot}}}$$

while for the detergent monomers ($\nu =1$) as well as detergent in micelles ($\nu >1$), we use

7 $${X}_{\nu }=\nu {N}_{\nu }/{N}_{{\text{tot}}}$$

As discussed by Bothe et al. (2023), we use an ideal solution model for the free energy of mixing, so that

8 $${G}_{{\text{mix}}}={\beta }^{-1}\left[{N}_{{\text{W}}}{\text{ln}}\frac{{N}_{{\text{W}}}}{\overline{N} }+\sum_{\nu }{N}_{\nu }{\text{ln}}\frac{{N}_{\nu }}{\overline{N} }+{N}_{{\text{P}}}{\text{ln}}\frac{{N}_{{\text{P}}}}{\overline{N} }+\sum_{\alpha }{N}_{\alpha }\frac{{N}_{\alpha }}{\overline{N} }\right]$$

where $\beta =1/{k}_{{\text{B}}}T$ with ${k}_{{\text{B}}}$ being Boltzmann´s constant and $T$ the absolute temperature and

9 $$\overline{N }={N}_{{\text{W}}}+{N}_{{\text{P}}}+{N}_{{\text{cos}}}+\sum_{\nu }{N}_{\nu }$$

Note that $\overline{N }$ is different from ${N}_{{\text{tot}}}$, since it constains a sum over ${N}_{\nu }$ rather than a sum over ${N}_{\nu }\nu$. The reason is that it is the number of independently moving particles such as whole micelles that matters in the free energy of mixing. In contrast, ${N}_{{\text{tot}}}$ counts the number of molecules and thus for a micelle, sums up all detergent molecules in the micelle. There is a subtle flaw in the formula for the free energy of mixing given by Bothe et al. (2023), since it expresses ${G}_{{\text{mix}}}$ in terms of the ratios ${N}_{{\text{i}}}/{N}_{{\text{tot}}}$ rather than ${N}_{{\text{i}}}/\overline{N }$. As we shall see, this is correct only for ${N}_{{\text{tot}}}\approx \overline{N }$, which is valid, if the detergent concentration is sufficiently low.

We consider interaction only between detergent monomers and PEG in the free energy of interaction ${G}_{{\text{int}}}$ in the spirit of a Bragg–Williams or mean field model:

10 $${G}_{{\text{int}}}=\frac{J}{\beta }\frac{{N}_{{\text{P}}}{N}_{1}}{\overline{N} }$$

where $J$ is a coupling constant (exchange parameter). Here again, $\overline{N }$ enters rather than ${N}_{{\text{tot}}}$.

To find the micellar size distribution, we have to minimize $G$ with respect to a variation of the numbers ${N}_{\nu }$ subject to the constraint given by Eq. (4). To this end, we define the new function

11 $$\mathcal{G}=G-\lambda \left(\sum_{\nu }{N}_{\nu }\nu -{N}_{{\text{det}}}\right)$$

with the Lagrange multiplier $\lambda$ and set $\partial \mathcal{G}/\partial {N}_{\nu }=0$ for all $\nu$. This procedure yields

12 $$\lambda = \frac{1}{\nu }\left( {\frac{\partial G}{{\partial N_{\nu } }}} \right)_{{T,P,N_{W} ,N_{P} ,\left\{ {N_{\alpha } } \right\},\left\{ {N_{\nu \prime } } \right\}}} {\text{for}}\; {\text{all}}\; \nu$$

from which it follows after some calculation that

13 $$-\beta \nu \left({\mu }_{\nu }^{0}-{\mu }_{1}^{0}\right)={\text{ln}}\frac{{N}_{\nu }{\overline{N} }^{\nu }}{{N}_{1}^{\nu }\overline{N} }+J\frac{{N}_{{\text{P}}}}{\overline{N} }\left(\left(\nu -1\right)\frac{{N}_{1}}{\overline{N} }-\nu \right)$$

We now make the simplifying assumption that ${N}_{{\text{tot}}}\approx \overline{N }$ and introduce the abbreviation

14 $$\xi =J\left(\left(\nu -1\right){X}_{1}-\nu \right)$$

Then

15 $$-\beta \nu \left({\mu }_{\nu }^{0}-{\mu }_{1}^{0}\right)={\text{ln}}\frac{{X}_{\nu }}{\nu {X}_{1}^{\nu }}+\xi {X}_{{\text{P}}}$$

which can be rearranged to give the micellar size distribution

16 $${X}_{\nu }=\nu {X}_{1}^{\nu }{\text{exp}}\left\{-\beta \nu \left({\mu }_{\nu }^{0}-{\mu }_{1}^{0}\right)-\xi {X}_{{\text{P}}}\right\}$$

For ${X}_{{\text{P}}}=0$, Eq. (16) is the same micellar size distribution as given by Bothe et al. (2023). For ${X}_{{\text{P}}}>0$, Eq. (16) shows the influence of PEG on the micellar size distribution due to a deviation from ideal solution behavior originating from ${G}_{{\text{int}}}$.

### Mass action model and definition of the critical micelle concentration

Since we do not know precisely, how the standard chemical potential ${\mu }_{\nu }^{0}$ depends on the number $\nu$, we assume a mass action model, in which the polydispersity of micelles is neglected and only one type of micelle with a fixed aggregation number $\nu =m$ is considered. This model allows for a molecular thermodynamic modeling of ${\mu }_{m}^{0}-{\mu }_{1}^{0}$ based on experimental information about the micelles as outlined by Bothe et al. (2023), who also discussed the limitations of this approach. If ${\text{D}}$ is a detergent monomer, then the mass action model implies that micelle formation can be described by the chemical equilibrium

17 $$m {\text{D}} \rightleftharpoons {{\text{D}}}_{m}$$

where ${{\text{D}}}_{m}$ is a micelle with aggregation number $m$. In this model, the equations derived above simplify significantly. If we set $y:={X}_{1}$ and $z:={X}_{m}$, we obtain from Eq. (16)

18 $$z=m{y}^{m}{\text{exp}}\left\{-m{g}_{{\text{mic}}}-\xi {X}_{{\text{P}}}\right\}$$

where now

19 $$\xi (y)=J\left(\left(m-1\right)y-m\right)$$

is a function of $y$ and

20 $${g}_{{\text{mic}}}=\beta \left({\mu }_{m}^{0}-{\mu }_{1}^{0}\right)$$

Furthermore, we define $x:={N}_{{\text{det}}}/{N}_{{\text{tot}}}$ as the total mole fraction of detergent, so that

21 $$x=y+z=y+m{y}^{m}{\text{exp}}\left\{-m{g}_{{\text{mic}}}-\xi (y){X}_{{\text{P}}}\right\}$$

The CMC can be determined by monitoring the fluorescence intensity $\phi (x)$ of a suitable dye as a function of the total detergent concentration (mole fraction) in the sample. The function $\phi (x)$ exhibits a sharp breaking point at the CMC. It has been shown by Bothe et al. (2023) that this breaking point coincides with that of the curve $y(x)$, which is the detergent monomer concentration as a function of total detergent concentration. Then, the CMC can be obtained from the condition

22 $${\left(\frac{{{\text{d}}}^{3}y}{{\text{d}}{x}^{3}}\right)}_{x={X}_{{\text{CMC}}}}=0$$

Here, ${X}_{{\text{CMC}}}$ is the total detergent mole fraction at the CMC. This mole fraction is related to the actual CMC in molarity units by

23 $${\text{CMC}}={c}_{{\text{tot}}}{X}_{{\text{CMC}}}$$

where ${c}_{{\text{tot}}}$ is the total molarity of the solution to be described below.

To exploit the condition (22), we define the function

24 $$f\left(x,y\right)=x-y-z(y)=x-y-m{y}^{m}{\text{exp}}\left\{-m{g}_{{\text{mic}}}-\xi (y){X}_{{\text{P}}}\right\}$$

and obtain from implicit differentiation

25 $$y^{\ \prime} = \frac{1}{{1 + \dot{z}}}$$

where a prime indicates a derivative with respct to $x$ and a dot a derivative with respect to $y$. As shown by Bothe et al. (2023), Eq. (22) then ultimatey leads to the condition

26 $$3\ddot{z}^{2} = \left( {1 + \dot{z}} \right)\dddot z$$

From this condition, it follows that at the breaking point

27 $$x=y+\frac{m\left(m-2\right)y+{a}_{2}{y}^{2}+{a}_{3}{y}^{3}+{a}_{4}{y}^{4}}{{m}^{2}\left(2m-1\right)+{b}_{1}y+{b}_{2}{y}^{2}+{b}_{3}{y}^{3} +{b}_{4}{y}^{4}}$$

with

28 $${a}_{2}=-3m\left(m-1\right)J{X}_{{\text{P}}}$$

29 $${a}_{3}=3m\left(m-1\right){\left(J{X}_{{\text{P}}}\right)}^{2}$$

30 $${a}_{4}=-{\left(m-1\right)}^{2}{\left(J{X}_{{\text{P}}}\right)}^{3}$$

31 $${b}_{1}=-2m\left(m-1\right)\left(4m+1\right)J{X}_{{\text{P}}}$$

32 $${b}_{2}=3m\left(m-1\right)\left(4m-1\right){\left(J{X}_{{\text{P}}}\right)}^{2}$$

33 $${b}_{3}=-8m{\left(m-1\right)}^{2}{\left(J{X}_{{\text{P}}}\right)}^{3}$$

and

34 $${b}_{4}=2{\left(m-1\right)}^{3}{\left(J{X}_{{\text{P}}}\right)}^{4}$$

For $J=0$, this result reduces to the equation

35 $$y=x\frac{{2m}^{2}-m}{{2m}^{2}-2}$$

found by Bothe et al. (2023). Unfortunately, Eq. (27) is too complicated for practical use. However, Bothe et al. (2023) found that for large $m$, $x\approx y$ at the breaking point corresponding to the traditional definition of the CMC. This result also follows from Eq. (27) for $m\to \infty$. To exploit this limit, we use Eq. (35) as an approximation for $y$, plug it into Eq. (21), and finally obtain for $m\to \infty$:

36 $${\text{ln}}{X}_{{\text{CMC}}}={g}_{{\text{mic}}}+\kappa {X}_{{\text{P}}}$$

where

37 $$\kappa =J\left({X}_{{\text{CMC}}}-1\right)$$

Since ${X}_{{\text{CMC}}}\ll 1$, we can consider $\kappa \approx -J$ practically as a constant. The derivation of Eq. (36) is described in the Supplementary Information (SI) along with a discussion of the involved approximations. Note that ${g}_{{\text{mic}}}$ is introduced in Eq. (20) as a function of the standard chemical potentials ${\mu }_{m}^{0}$ and ${\mu }_{1}^{0}$, which according to the modeling of ${g}_{{\text{mic}}}$ by Bothe et al. (2023) should be interpreted as reference states pertaining to an infinite dilution of micelles and monomers, respectively, in buffer (not pure water). Thus, ${g}_{{\text{mic}}}$ refers to zero PEG concentration, i.e., ${g}_{{\text{mic}}}={\text{ln}}{X}_{{\text{CMC}}}(0)$ with ${X}_{{\text{CMC}}}(0)$ being the total mole fraction of detergent at the CMC in the absence of PEG. An alternative view is to interpret the additional term $\kappa {X}_{{\text{P}}}$ in Eq. (36) as a shift of the standard chemical potential of the monomers, i.e., ${g}_{{\text{mic}}}\left({X}_{{\text{P}}}\right)=\beta \left({\mu }_{m}^{0}-{\mu }_{1}^{0}\left({X}_{{\text{P}}}\right)\right)=\beta \left({\mu }_{m}^{0}-\left[{\mu }_{1}^{0}\left(0\right)-{k}_{{\text{B}}}T\kappa {X}_{{\text{P}}}\right]\right)$. This view is useful for discussions of CMC shifts in terms of equilibrium constants (see below), but implies a change of the standard state.

From Eq. (36), we obtain

38 $${\text{ln}}\frac{{X}_{{\text{CMC}}}({c}_{{\text{P}}})}{{X}_{{\text{CMC}}}(0)}=\kappa \frac{{c}_{{\text{P}}}}{{c}_{{\text{tot}}}}$$

Here, ${c}_{{\text{P}}}$ is the PEG concentration. In the following, we will also indicate the presence of PEG by $\chi$, which is the PEG concentration given in % (w/v) related to the PEG molarity ${c}_{{\text{P}}}$ in a simple way (see below). If we denote by ${c}_{{\text{tot}}}(\chi )$ and ${c}_{{\text{P}}}(\chi )$ the total molarity of the solution and the PEG molarity, respectively, as a function of $\chi$, we obtain the following relationship:

39 $${c}_{{\text{tot}}}\left(\chi \right) {\text{ln}}\frac{{X}_{{\text{CMC}}}(\chi )}{{X}_{{\text{CMC}}}(0)}=\kappa {c}_{{\text{P}}}\left(\chi \right)={\kappa }^{*} \chi$$

Here, $\kappa$ is a dimensionless quantity, while ${\kappa }^{*}$ relates mol L ^−1^ to % (w/v).

## Experimental methods

Experiments were performed by D. DiFiore in the course of our earlier work (Müh et al. 2015). Detergents were purchased from Glycon (Luckenwalde, Germany), all other chemicals from Sigma-Aldrich, and used without further purification. The investigated detergents are *n*-alkyl-β-D-maltosides with the number of carbon atoms in the alkyl chain ranging from $n=10$ to $n=12$. (The “*n*” in “*n*-alkyl” refers to “normal,” i.e., nonbranched alkyl chains.) For convenience, these detergents are referred to as DM (decyl maltoside), UDM (undecyl maltoside), and DDM (dodecyl maltoside). All experiments were performed using a buffer system suitable for the crystallization of PSII (Loll et al. 2005). The buffer contains 100 mM piperazine-1,4-bis-(2-ethanesulfonic acid) (PIPES), adjusted to pH 7.0 with NaOH and 5 mM CaCl_2_. The molar masses of the buffer components are ${M}_{{\text{CaCl}}2}=110.98 \, {\text{g}} \, {{\text{mol}}}^{-1}$ for CaCl_2_ and ${M}_{{\text{PIP}}}=300.44 \, {\text{g}} \, {{\text{mol}}}^{-1}$ for PIPES. In the latter case, the protonation probability at pH 7.0, determined by using the Henderson–Hasselbalch equation based on p*K*_*a*_ = 6.76, was considered in the determination of the molar mass (Bothe et al. 2023). The CMC was determined by exploiting the fluorescence enhancement upon binding to micelles of the dye 8-anilinonaphtalene-1-sulfonate (ANS) as outlined in the literature (De Vendittis et al. 1981; Abuin et al. 1997). Fluorescence experiments were performed with 1.5 mL samples containing up to 10 µM ANS and the fluorescence recorded with a Horiba Jobin Yvon FluoroMax-2 spectrometer in the range between 460 and 530 nm with an integration time of 0.5 s and excitation at 370 nm. Titrations were carried out by adding 2 µL aliquots of a detergent stock solution to the 1.5 mL sample and taking into account the dilution in the determination of the total detergent concentration. All experiments were performed at room temperature. The titration curves were analyzed by a graphical extrapolation procedure to determine the breaking point at the CMC as described before (Müh et al. 2015; Bothe et al. 2023). The densities of the buffer solutions were determined by using a Mettler-Toledo DA-100 M digital density meter.

## Results

### Characterization of PEG and total molarity

Poly(ethylene glycol) is a polyether compound with the chemical formula H–(O–CH_2_–CH_2_)_*p*_–OH referred to as PEG ${M}_{{\text{r}}}$, when the relative molecular weight ${M}_{{\text{r}}}$ lies in the range from 200 to 35.000 (larger polymers are referred to as poly(ethylene oxide)). For any PEG, the given value of ${M}_{{\text{r}}}$ is only an average, and the concentrations are often given in % (w/v) denoted here by $\chi$. In order to obtain PEG molarities, we have to relate the molar mass ${M}_{{\text{P}}}$ to the number $p$ of oxyethylene (OE) units according to

40 $${M}_{{\text{P}}}=p {M}_{{\text{OE}}}+{M}_{{{\text{H}}}_{2}{\text{O}}}$$

with ${M}_{{\text{OE}}}=44.053 \, {\text{g}} \, {{\text{mol}}}^{-1}$ and ${M}_{{{\text{H}}}_{2}{\text{O}}}=18.015 \, {\text{g}} \, {{\text{mol}}}^{-1}$. For any given average value of ${M}_{{\text{r}}}$, we can then determine a representative average value of $p$ for the given type of PEG:

41 $$p=\frac{{M}_{{\text{P}}}-{M}_{{{\text{H}}}_{2}{\text{O}}}}{ {M}_{{\text{OE}}}}=\frac{{M}_{{\text{r}}} \, {\text{g}} { \, {\text{mol}}}^{-1}-{M}_{{{\text{H}}}_{2}{\text{O}}}}{ {M}_{{\text{OE}}}}$$

For PEG types of interest in the present work, the values of $p$ are listed in Table 1. We note that the relationship ${M}_{{\text{P}}}={M}_{{\text{r}}} \, {\text{g}} \, {{\text{mol}}}^{-1}$ is only approximately valid due to a redefinition of SI base units from 2019, but the deviation is practically negligible.

**Table 1:** Relative molecular weight ${M}_{{\text{r}}}$ and representative number of OE units $p$ for various PEG types

${M}_{{\text{r}}}$	$p$	$10 p/{M}_{{\text{r}}}$
300	6.401	0.2134
400	8.761	0.2168
550	12.076	0.2196
1000	22.291	0.2229
2000	44.991	0.2250
3350	75.636	0.2258
4000	90.391	0.2260
6000	135.791	0.2263
8000	181.190	0.2265

Since $\chi$ is given in % (w/v) (corresponding to g/mL × 100), the PEG molarity (in mol/L) is obtained as

42 $${c}_{{\text{P}}}=\frac{10}{{M}_{{\text{P}}}}\chi$$

Also, from Eq. (39), we have

43 $${\kappa }^{*}=\frac{10}{{M}_{{\text{P}}}}\kappa$$

The molar concentration of OE units is given by

44 $${c}_{{\text{OE}}}=p {c}_{{\text{P}}}=\frac{10 p}{{M}_{{\text{P}}}}\chi =\frac{10 p}{{M}_{{\text{r}}} \, {\text{g}} \, {{\text{mol}}}^{-1}}\chi$$

**Fig. 1:**
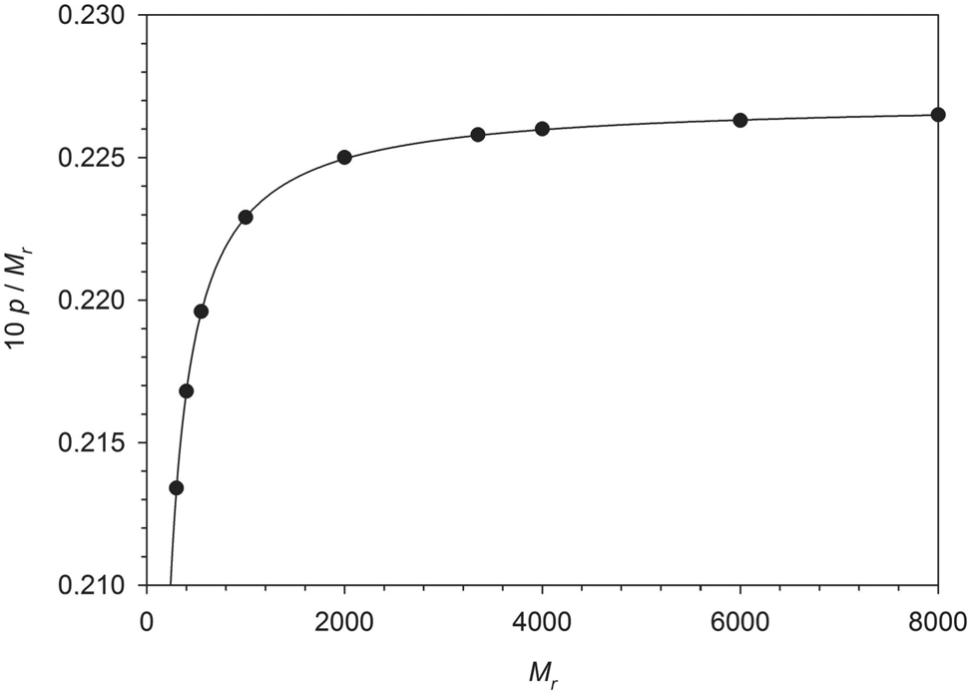
Dependence of the quantity $10 p/{M}_{{\text{r}}}$ determining the ratio between the molar concentration of oxyethylene (OE) units ${c}_{{\text{OE}}}$ and the PEG concentration $\chi$ in % (w/v) according to Eq. (44) on the relative molecular weight ${M}_{{\text{r}}}$ of the PEG chain. The solid curve is the function $(10/44.053)(1-18.015/{M}_{{\text{r}}})$. Figure and fit made with SigmaPlot 13 (© 2014 Systat Software Inc.)

Thus, ${c}_{{\text{OE}}}$ is proportional to $\chi$ with the proportionality constant depending on the relative molecular weight as shown in Fig. 1. Apparently, the dependence levels off at higher relative molecular weights, since the influence of the end groups of the polymer becomes smaller.

**Fig. 2:**
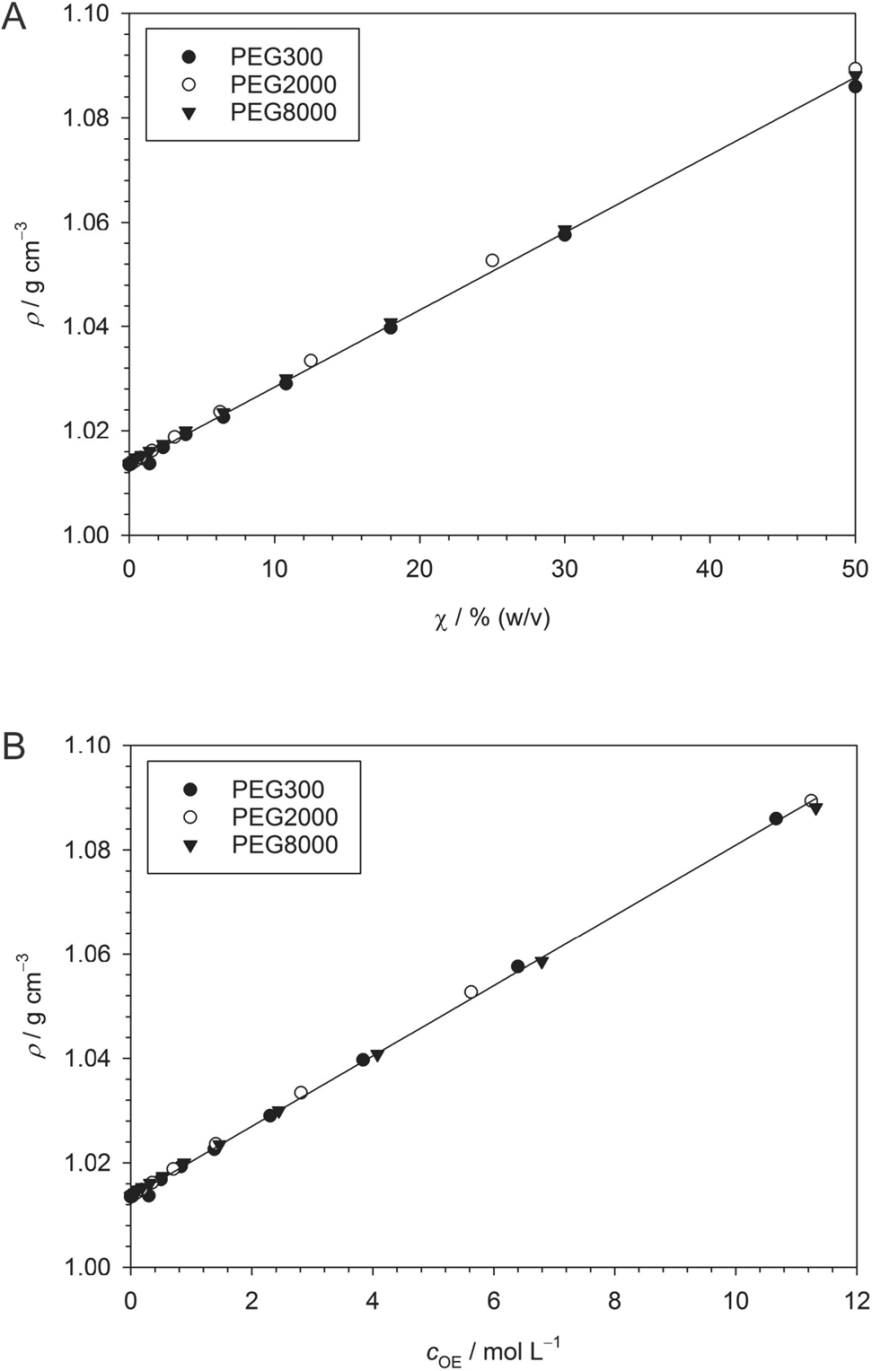
Dependence of the density of PEG solutions in aqueous 100 mM PIPES (pH 7.0), 5 mM CaCl_2_ on the PEG concentration for PEG300, PEG2000, and PEG8000. **A** Dependence on $\chi$. The straight line is a linear fit to all data with slope 0.0015 ± 8.5 × 10^−6^ g cm^−3^ / % (w/v) and the intercept fixed to 1.0135 g cm^−3^ (*R*^2^ = 0.9978). **B** Dependence on ${c}_{{\text{OE}}}$. The straight line is a linear fit to all data with slope 0.0067 ± 2.2 × 10^−5^ g cm^−3^ /mol L^−1^ and the intercept fixed to 1.0135 g cm^−3^ (*R*^2^ = 0.9993). Figures and fits made with SigmaPlot 13 (© 2014 Systat Software Inc.)

The total molarity of the solution is given by

45 $${c}_{{\text{tot}}}=\frac{\rho -{c}_{{\text{P}}}{M}_{{\text{P}}}}{{M}_{{\text{wat}}}}+{c}_{{\text{P}}}+{k}_{{\text{cos}}}=\frac{\rho }{{M}_{{\text{wat}}}}+{c}_{{\text{P}}}\left(1-\frac{{M}_{{\text{P}}}}{{M}_{{\text{wat}}}}\right)+{k}_{{\text{cos}}}=\frac{\rho }{{M}_{{\text{wat}}}}+{k}_{{\text{cos}}}+\chi 10\left(\frac{1}{{M}_{{\text{P}}}}-\frac{1}{{M}_{{\text{wat}}}}\right)$$

with

46 $${k}_{{\text{cos}}}={c}_{{\text{PIP}}}+{c}_{{\text{CaCl}}2}-\frac{{c}_{{\text{PIP}}}{M}_{{\text{PIP}}}+{c}_{{\text{CaCl}}2}{M}_{{\text{CaCl}}2}}{{M}_{{\text{wat}}}}$$

where $\rho$ is the density (in g dm^−3^) of the solution. ${M}_{{\text{wat}}}$, ${M}_{{\text{P}}}$, ${M}_{{\text{PIP}}}$, and ${M}_{{\text{CaCl}}2}$ are, respectively, the molar masses of water, PEG, buffer, and CaCl_2_, while ${c}_{{\text{P}}}$, ${c}_{{\text{PIP}}}$, and ${c}_{{\text{CaCl}}2}$ are the corresponding molarities. (The molarity of water is given by $(\rho -{c}_{{\text{P}}}{M}_{{\text{P}}}-{c}_{{\text{PIP}}}{M}_{{\text{PIP}}}-{c}_{{\text{CaCl}}2}{M}_{{\text{CaCl}}2})/{M}_{{\text{wat}}}$). Here, we have neglected the contributions from ANS and detergent. According to Eq. (45), we need the density of the solution to determine the total molarity, which in turn is needed to relate mole fractions to molar concentrations. Thus, we determined the density of our buffer system as a function of PEG concentration for PEG300, PEG2000, and PEG8000 (Fig. 2).

**Fig. 3:**
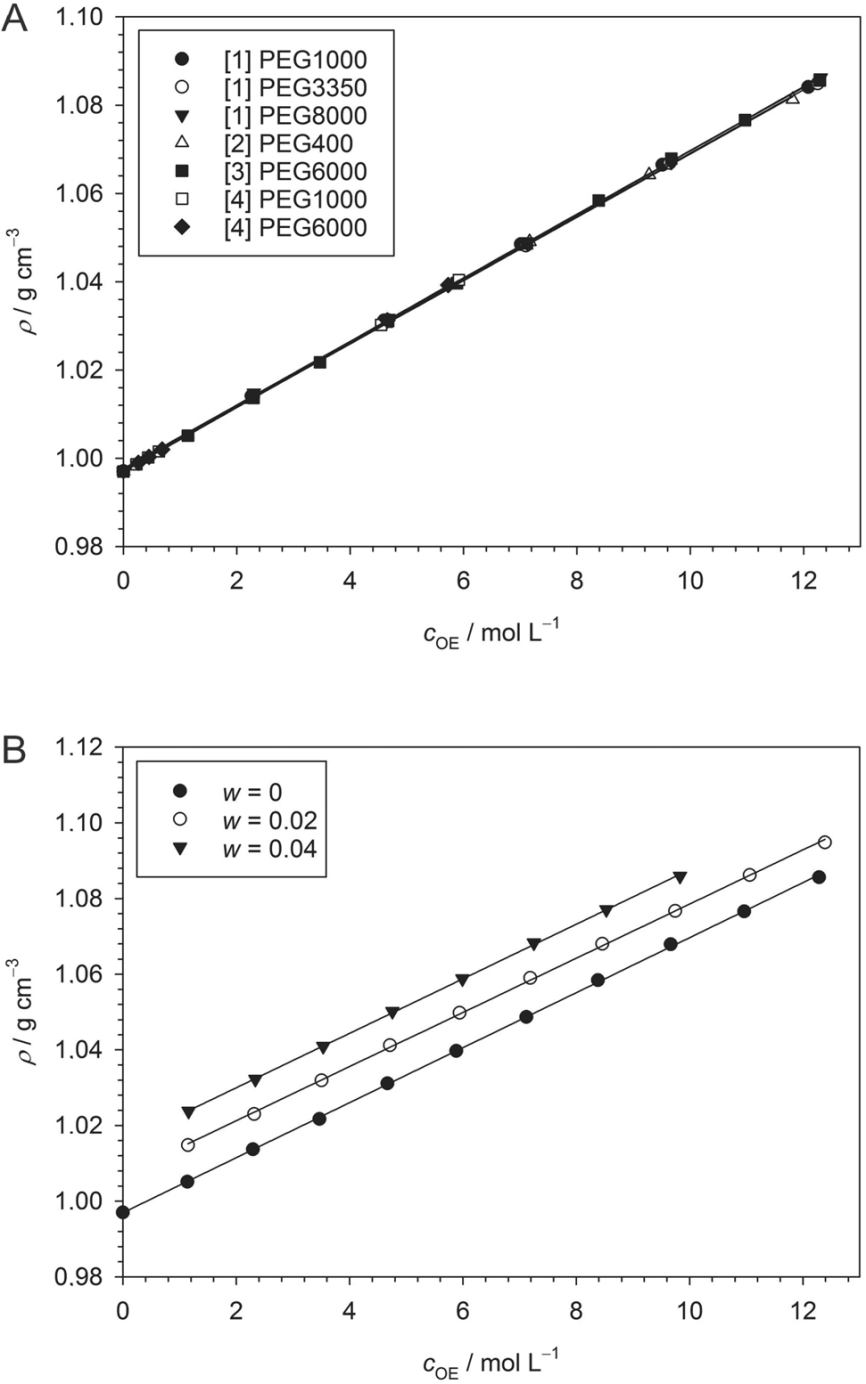
Dependence of the density of aqueous PEG solutions compiled from the literature. **A** Dependence on ${c}_{{\text{OE}}}$ in water at 25 °C computed from data published in [1] (González-Tello et al. 1994), [2] (Müller and Rasmussen 1991), [3] (Regupathi et al. 2009), and [4] (Zafarani-Moattar et al. 1995). The straight lines are linear fits to the individual data sets with slopes in the range 0.0073 ± 0.0001 g cm^−3^/mol L^−1^ and intercepts 0.9973 ± 0.0004 g cm^−3^. **B** Dependence on ${c}_{{\text{OE}}}$ of PEG6000 in aqueous solutions of triammonium citrate with the indicated mass fraction *w* of citrate at 25 °C (Regupathi et al. 2009). The straight lines are linear fits to the individual data sets with slopes in the range 0.0072 ± 5 × 10^−5^ g cm^−3^/mol L^−1^ and intercepts 0.9970 ± 0.0002 g cm^−3^ (*w* = 0), 1.0070 ± 0.0003 g cm^−3^ (*w* = 0.02), and 1.0156 ± 0.0003 g cm^−3^ (*w* = 0.04). Figures and fits made with SigmaPlot 13 (© 2014 Systat Software Inc.)

In the absence of PEG, our buffer has a density of $\rho =1.0135 \, {\text{g}} \, {{\text{cm}}}^{-3}$. Addition of PEG causes a linear increase of the density as a function of either $\chi$ (Fig. 2A) or ${c}_{{\text{OE}}}$ (Fig. 2B) that is practically independent of the relative molecular weight of the PEG type. From linear regression, we obtain

47 $$\rho \left(\chi \right)=1.0135 \, {\text{g}} \, {{\text{cm}}}^{-3}+0.0015 \frac{{\text{g}} \, {{\text{cm}}}^{-3}}{\%({\text{w}}/{\text{v}})} \chi$$

and

48 $$\rho \left({c}_{{\text{OE}}}\right)=1.0135 \, {\text{g}} { \, {\text{cm}}}^{-3}+0.0067 \frac{{\text{g}} \, {{\text{cm}}}^{-3}}{{\text{mol}} \, {{\text{L}}}^{-1}} {c}_{{\text{OE}}}$$

(see caption to Fig. 2 for error margins). These results are in good agreement with literature data (Fig. 3). A compilation of density data for various PEG types solved in pure water at 25 °C (Fig. 3A) results in the relationship

49 $$\rho \left({c}_{{\text{OE}}}\right)=0.9973 \, {\text{g}} { \, {\text{cm}}}^{-3}+0.0073 \frac{{\text{g}} \, {{\text{cm}}}^{-3}}{{\text{mol}} { \, {\text{L}}}^{-1}} {c}_{{\text{OE}}}$$

for all PEG types, confirming the independence of the relative molecular weight. Regupathi et al. (2009) measured the density of PEG6000 solutions in aqueous triammonium citrate (Fig. 3B). Their data result in practically the same slope of $\rho$ as a function of ${c}_{{\text{OE}}}$ with only the intercept changing according to the mass fraction of citrate. We thus conclude that in general, the density of PEG solutions may be computed to a good approximation from

50 $$\rho \left({c}_{{\text{OE}}}\right)={\rho }_{0}+0.007 \frac{{\text{g}} { \, {\text{cm}}}^{-3}}{{\text{mol}} \, {{\text{L}}}^{-1}} {c}_{{\text{OE}}}$$

irrespective of the relative molecular weight of PEG, where ${\rho }_{0}$ is the density of the aqueous solution system at zero PEG concentration.

If we plug Eq. (48) (with $\rho$ given in g dm^−3^) into Eq. (45) and take into account Eq. (44) and ${k}_{{\text{cos}}}$ in Eq. (46), we obtain

51 $${c}_{{\text{tot}}}=54.665\frac{{\text{mol}}}{ {\text{L}}} -2.073 {c}_{{\text{OE}}}$$

Equation (51) is derived and discussed in the SI. This linear relationship between ${c}_{{\text{tot}}}$ and ${c}_{{\text{OE}}}$ is valid for all PEG types with the given buffer in the range $0\le \chi \le 50\ \%\ ({\text{w}}/{\text{v}})$.

### PEG-induced CMC shift

**Fig. 4:**
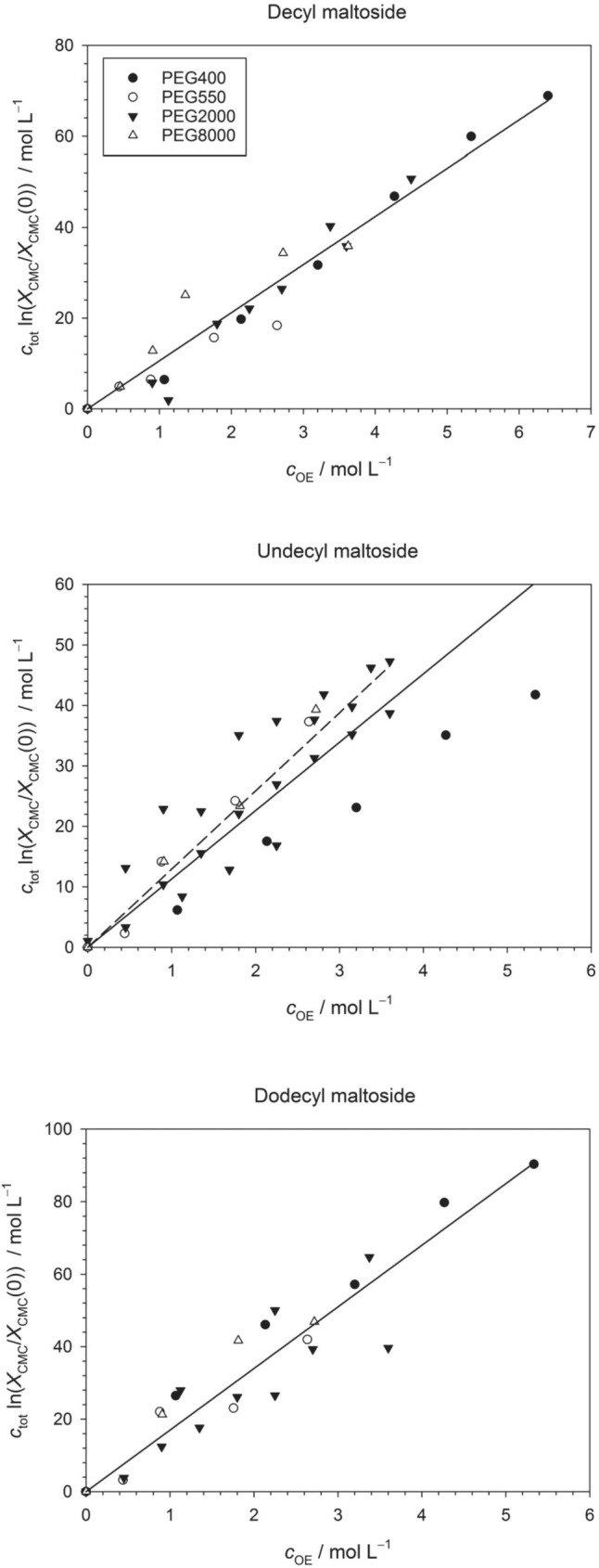
Plots of ${c}_{{\text{tot}}} {\text{ln}}\left({X}_{{\text{CMC}}}({c}_{{\text{OE}}})/{X}_{{\text{CMC}}}(0)\right)$ (left side of Eq. (52)) versus ${c}_{{\text{OE}}}$ for the three investigated alkyl maltosides and the indicated PEG types. The slope of the plot is $\kappa /p$. The solid lines are linear regressions for all data points with zero intercept and the following slopes: DM, 10.6 ± 0.3 (*R*^2^ = 0.9515); UDM 11.3 ± 0.6 (*R*^2^ = 0.7567); DDM, 17.0 ± 0.7 (*R*^2^ = 0.9017). The dashed line in the case of UDM is a fit with the data points for PEG400 excluded, yielding a slope of 12.9 ± 0.5 (*R*^2^ = 0.8704). Figures and fits made with SigmaPlot 13 (© 2014 Systat Software Inc.)

The influence of PEG on the CMC of alkyl maltosides was investigated by using the ANS-fluorescence method as outlined in the literature (De Vendittis et al. 1981; Abuin et al. 1997; Müh et al. 2015; Bothe et al. 2023) for DM, UDM, and DDM combined with PEG400, PEG550, PEG2000, and PEG8000. Data for PEG2000 published earlier (Müh et al. 2015) were included, but supplemented with additional data points. To analyze the PEG-induced CMC shift, we recast Eq. (39) in terms of ${c}_{{\text{OE}}}$ as

52 $${c}_{{\text{tot}}} {\text{ln}}\frac{{X}_{{\text{CMC}}}({c}_{{\text{OE}}})}{{X}_{{\text{CMC}}}(0)}=\frac{\kappa }{p} {c}_{{\text{OE}}}$$

where ${c}_{{\text{tot}}}$ is given by Eq. (51) and the dependence of ${c}_{{\text{tot}}}$ on ${c}_{{\text{OE}}}$ is also taken into account in the computation of ${X}_{{\text{CMC}}}({c}_{{\text{OE}}})$ from the experimental CMC values based on Eq. (23). Plots of the left side of Eq. (52) versus ${c}_{{\text{OE}}}$ are shown in Fig. 4 (see SI, Table S1 for the original CMC data).

Although there is some scattering in the data, a linear correlation is clearly visible. According to the values of *R*^2^ originating from linear regression (see caption to Fig. 4), the best correlation is obtained for DM and the worst for UDM. Specifically, the data for DM suggest that all PEG types induce the same CMC shift (i.e., change of ${c}_{{\text{tot}}} {\text{ln}}\left({X}_{{\text{CMC}}}({c}_{{\text{OE}}})/{X}_{{\text{CMC}}}(0)\right)$) irrespective of the relative molecular weight of PEG, when analyzed as a function of the concentration of OE units. To substantiate this suggestion, we also performed linear regressions for each PEG type separately (SI Table S2, Figs. S1–S3). These fits yield similar slopes for all PEG types with the values for one detergent deviating from each other by not more than 3 units except for UDM/PEG400, which is the most significant outlier. It is also noteworthy that for each detergent, there are two PEG types that yield essentially the same slope, but these are different PEG types for each detergent (i.e., PEG400 and PEG2000 for DM, PEG550 and PEG8000 for UDM as well as PEG550 and PEG2000 for DDM). For none of the detergents, there is a clear correlation between the slope and the relative molecular weight of PEG. Some of the PEG2000 data have been published before (Müh et al. 2015). If we analyze these data separately, we obtain slopes of 9.9 ± 0.4 for DM, 11.4 ± 0.2 for UDM, and 12.5 ± 0.6 for DDM. These data demonstrate that the mere addition of data points from independent measurements can change the slope by up to about 3 units. Apparently, there is a statistical spread in the data, which might be reduced by additional measurements. Thus, the present data suggest that the CMC shift is determined by ${c}_{{\text{OE}}}$ and is independent of ${M}_{{\text{r}}}$, but further data should be generated to validate this hypothesis.

**Fig. 5:**
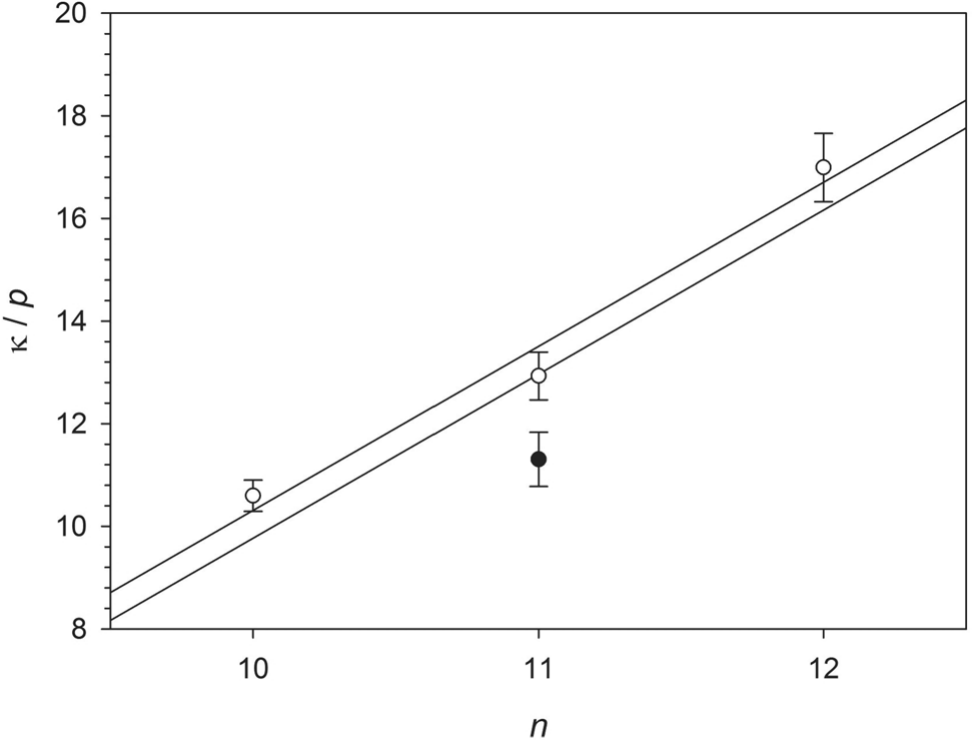
Change of the slope $\kappa /p$ with the number of carbon atoms in the alkyl chain of the detergent $n$. Closed symbols refer to slopes obtained from fits including all available data, whereas open symbols (covering the closed symbols for $n=10$ and $n=12$) refer to slopes obtained by omitting the UDM/PEG400 data. The error bars refer to the uncertainties of the slopes inferred from linear regression and given in the caption to Fig. 4. The straight lines shown both have a slope of 3.2. Figure and fits made with SigmaPlot 13 (© 2014 Systat Software Inc.)

There is a clear tendency of the $\kappa /p$ -values to increase when going from DM to DDM, i.e., with increasing number of carbon atoms in the alkyl chain $n$ (Fig. 5). If all data are included in the analysis, there seems to be a nonlinear relationship beween $\kappa /p$ and $n$ (closed symbols in Fig. 5 partially covered by open symbols). A linear relationship within the fitted error margins of $\kappa /p$ is obtained, if the UDM/PEG400 data set is omitted (open symbols in Fig. 5). For both cases, a linear fit yields a slope of 3.2 (see straight lines in Fig. 5).

## Discussion

The formation of micelles is a complicated self-assembly process yielding micelles with a size distribution that may be inferred from Eq. (16). However, modeling the dependence of the standard chemical potential ${\mu }_{\nu }^{0}$ on the agregation number $\nu$ is challenging (Nagarajan and Ruckenstein 1991). In order to gain a simpler, chemically intuitive understanding, one often invokes a mass action model, in which the micelle formation is regarded as a simple chemical equilibrium as shown in Eq. (17), where all the micelles have the same size with aggregation number $m$. The associated equilibrium constant $K$ is given by (Bothe et al. 2023)

53 $$K=\frac{z}{m{y}^{m}}={e}^{-m{g}_{{\text{mic}}}}={e}^{-\Delta {G}^{0}/{k}_{{\text{B}}}T}$$

where $\Delta {G}^{0}={k}_{{\text{B}}}Tm{g}_{{\text{mic}}}$ is the standard micellization (”reaction”) free energy. Note that $K$ is formulated in terms of dimensionless quantities as it should be (Özcan 2022). In the limit of large $m$, a simple relationship between ${g}_{{\text{mic}}}$ and the CMC in mole fraction units, ${X}_{{\text{CMC}}}$, holds, so that

54 $${\text{ln}}K=-m{g}_{{\text{mic}}}=-m{\text{ln}}{X}_{{\text{CMC}}}$$

Therefore, in this limit, ${X}_{{\text{CMC}}}$ can be interpreted as a measure for the equilibrium constant of micelle formation. Note that if ${X}_{{\text{CMC}}}$ increases, $K$ decreases, i.e., an increase of the CMC indicates a shift of the equilibrium in Eq. (17) towards detergent monomers. Such a shift is caused by PEG.

The standard micellization free energy can be written in terms of the CMC as

55 $$\Delta {G}^{0}=-{k}_{{\text{B}}}T{\text{ln}}K={k}_{{\text{B}}}Tm {\text{ln}}\frac{{\text{CMC}}}{{c}_{{\text{tot}}}}$$

Our fitting relationship in Eq. (52) can be recast as

56 $${\text{ln}}\frac{{\text{CMC}}}{{c}_{{\text{tot}}}}- {\text{ln}}\frac{{\text{CMC}}\left(0\right)}{{c}_{{\text{tot}}}\left(0\right)}=\frac{\kappa }{p} \frac{{c}_{{\text{OE}}}}{{c}_{{\text{tot}}}}$$

where $"(0)"$ stands for zero PEG concentration. It is now obvious that the change in standard micellization free energy due to PEG, $\mathrm{\Delta \Delta }{G}^{0}(c_{\text{OE}} )$, with zero PEG concentration as reference, is given by

57 $$\mathrm{\Delta \Delta }{G}^{0}\left({c}_{{\text{OE}}}\right)=\Delta {G}^{0}\left({c}_{{\text{OE}}}\right)-\Delta {G}^{0}(0)={k}_{{\text{B}}}Tm \frac{\kappa }{p} \frac{{c}_{{\text{OE}}}}{{c}_{{\text{tot}}}}$$

Thus, the change in standard micellization free energy is proportional to the mole fraction of OE units with proportionality constant ${k}_{{\text{B}}}Tm\kappa /p$.

Before proceeding with the interpretation of $\kappa /p$, we have to discuss the evidence that it is independent of the PEG type. While it is well known that PEG increases the CMC of nonionic detergents (Aoudia and Zana 1998; Hitscherich et al. 2000; Blouwolff and Fraden 2007; Santonicola et al. 2007), we show here—to our knowledge for the first time—by a systematic variation of the PEG molecular weight and the alkyl chain length of the detergent, that the CMC shift is independent of the molecular weight and determined by the concentration of OE units. The evidence comes in particular from those cases, where two PEGs with significantly different molecular weights cause the same shift, i.e., PEG400 and PEG2000 for DM, PEG550 and PEG8000 for UDM as well as PEG550 and PEG2000 for DDM. For each detergent, the other PEG types cause similar shifts with slopes differing by about 3 units with the exception of UDM/PEG400 showing a somewhat larger deviation. We interpret these deviations as being statistical in nature as is supported by a comparison of our earlier PEG2000 data (Müh et al. 2015) with the extended data set studied here. The extension of the PEG2000 data sets causes a change of the slopes similar to the deviations between slopes for different PEG types. Thus, the problem of scattering in the data might be reduced by measuring further data points in future work. In addition, there might be systematic errors that could be reduced. Possible strategies are a strict temperature control, a careful analysis of possible pipetting errors, further purification of chemicals before use, and the development of a fitting procedure for the original fluorescence titration curves to get rid of the graphical extrapolation procedure.

We also plotted ${\text{ln}}({\text{CMC}}/{\text{CMC}}(0))$ versus $\chi$ (see SI, Figs. S4–S6) as done in our earlier work (Müh et al. 2015). The slopes obtained from linear regression of these plots show the same trends as those as a function of ${c}_{{\text{OE}}}$ (SI, Table S3), so that the relation between $\chi$ and ${c}_{{\text{OE}}}$ (Fig. 1) can be ruled out as a major source of a different behavior of PEG types. The two ways of describing the CMC shift are essentially equivalent. However, the description based on Eq. (52) has the advantage of yielding the slope $\kappa /p$, which on the basis of Eq. (57) is a direct measue of how PEG changes the standard micellization free energy. We note that an alternative approach is to plot ${\text{ln}}\left({X}_{{\text{CMC}}}/{X}_{{\text{CMC}}}(0)\right)$ as a function of ${c}_{{\text{OE}}}/{c}_{{\text{tot}}}$, yielding practically the same slopes as Eq. (52). The reason for the equivalence of these presentations is that deviations from linearity are small compared to the scattering of the data.

The dependence of the CMC shift on ${c}_{{\text{OE}}}$ has an interesting implication, as any PEG is equivalent to independent OE units as far as the effect on the CMC is concerned. However, a certain OE concentration of, say PEG400 is, of course, different from the same OE concentration of PEG8000, since the number of covalently connected OE units $p$ is different and so is the number of independently moving molecules. This difference has consequences for the entropy of mixing and the conformational entropy of the polymer. Our data suggest that the CMC of alkyl maltosides is not affected by such entropy effects or that their influence is so small that it is hidden in the scattering of the data.

Assuming from now on that $\kappa /p$ is indeed independent of the molecular weight of the PEG chain, we come to the interesting question of how it depends on the detergent. We have studied three detergents with the same head groups (maltose), but differing in the number $n$ of carbon atoms in the alkyl tail. The idea behind such studies is to uncover contributions from the hydrophobic effect, i.e., the tendency of hydrophobic solutes to aggregate in aqueous solution, which is the major driving force for detergent self-assembly. According to molecular thermodynamic models (Nagarajan and Ruckenstein 1991; Müh et al. 2015; Bothe et al. 2023), $\Delta {G}^{0}$ is dominated by terms that depend linearly on $n$. As a consequence, the CMC depends exponentially on $n$, so that, for example, the CMC of DM is by one order of magnitude larger than that of DDM (see, e.g., the first row in Table S1). Consequently, any co-solute that influences the hydrophobic effect is expected to have an effect on the CMC that scales with the alkyl chain length. Indeed, we observe such a scaling in our data for PEG similar to what we found earlier for PEG2000 (Müh et al. 2015). However, a linear scaling of $\kappa /p$ with $n$ is only obtained, if the UDM/PEG400 data set is omitted (Fig. 5). Therefore,we conclude that more experimental data have to be collected, before a definitive conclusion about this scaling can be drawn.

Irrespective of errors, it is evident that PEG increases the CMC of alkyl maltosides in a buffer suitable for PSII crystallization. In our thermodynamic model, we considered only interactions between PEG and detergent monomers (see Eq. (10)). This was done here primarily to keep the mathematics controllable. Clearly, for a realistic description of the PEG effect, the model has to be extended. In the SI, we present a draft model that includes PEG–micelle interactions. As is obvious from the derivation, the mathematics becomes more complicated. In particular, the evaluation of the ratio between total detergent and monomer concentration at the CMC based on Eq. (22) is bedeviled by the fact that the exponent in the detergent mass balance becomes a function of $x$ and $y$ (see SI, Eq. (S35)). With the assumption that Eq. (35) (see above in the main text) is still a good approximation, we arrive at a solution that basically states that $\kappa$ has to be reinterpreted, if we include PEG–micelle interactions: Instead of $\kappa \approx -J$, it is now given by SI Eq. (S41), i.e., determined by the *difference* in the interaction of PEG with micelles and detergent monomers. Our analysis of the experimental data remains valid with this new interpretation of $\kappa$. However, we cannot push the analysis any further without additional independent information about the two coupling constants ${J}_{m}$ and ${J}_{1}$ (where ${J}_{1} = J$, see SI). We do not obtain this kind of information from our experiments.

The extended model presented in the SI is still incomplete as it does not allow for PEG–detergent aggregation. For example, PEG chains could attach to micelles and form aggregates with a more complicated composition and a different number of detergent molecules. Mixtures of neutral polymers and detergents in water have been widely studied in the past (Anthony and Zana 1994; Aoudia and Zana 1998). In particular, Zana and coworkers studied *inter alia* the influence of PEG20000 ($p=453.590$) on the aggregation number of DDM (as well as of C_12_E_8_, a different type of nonionic detergent). The polymer concentration was ~ 2% (w/v) corresponding to ${c}_{{\text{OE}}}=0.45\ \mathrm{mol }\ {{\text{L}}}^{-1}$. They found no measurable influence on the aggregation number. This result was interpreted as a negligible interaction between PEG and micelles and prompted us to start with the simple mass action model oulined above, which cannot account for changes of $m$. However, ~ 2% is a rather low value, and it would be of interest to determine the aggregation number at higher PEG concentrations. We note that modeling the PEG effect on the aggregation number is challenging as it would require the determination of model parameters that characterize the polydispersity of micelles. In the absence of PEG, it was found that the aggregation number of DDM has a significant rms deviation of about 40, which is rather independent of the detergent concentration (Warr et al. 1986). On the basis of the presently available data, we speculate that the effect of PEG on the aggregation number of alkyl maltosides is not larger than this rms deviation. Note also that different methods to determine the aggregation number yield different values: For DDM, small-angle X-ray scattering data suggest $m=140\pm 10$ (Lipfert et al. 2007), whereas fluorescence quenching experiments give $m=125\pm 10$ (Aoudia and Zana 1998), $m=138+3$ (Tummino and Gafni 1993), or $m=111\pm 10$ (Warr et al. 1986). The result is an overall uncertainty of 50, which is even larger than the rms deviation determined by fluorescence quenching techniques (Warr et al. 1986). Having said this, we also note that Warr et al. (1986) found the mean aggegation number of DDM micelles to be somewhat smaller in the vicinity of the CMC (80) than at significantly higher total detergent concentrations (111). This phenomenon is not yet properly understood.

Even if the aggregation number is not affected by PEG, this does not necessarily mean that there is no interaction between PEG and micelles. In fact, static light scattering studies provided evidence for such interactions, resulting in an effective weak attraction between micelles (second virial coefficient) that is relevant to understand the formation of type-II membrane protein crystals (Hitscherich et al. 2000). However, even the extended model presented in the SI is too simple to account for micelle–micelle interactions. We are working on this problem, but it is beyond the scope of the present report.

Meanwhile, we work with the simple model, in which PEG interacts only with detergent monomers. An alternative view of this model is to say that PEG enhances the solubility of the alkyl chains in the aqueous phase. It is known that PEG as a co-solute increases the solubility in water of nonpolar compounds such as naphtalene and biphenyl (Okubo and Ise 1969), argon, methane, ethane, and propane (King 1991), or other poorly water-soluble compounds like the barbiturate temazepan (Van den Mooter et al. 1998) or the anti-inflammatory drug valdecoxib (Liu et al. 2005). More similar to detergents are *n*-alkyl alcohols with the number of carbon atoms between 1 and 5, which were investigated in aqueous biphasic systems composed of PEG and salts (Willauer et al. 2002). The authors characterized the partitioning of the alcohols between the two phases by the transfer free energy of a methylene group, $\Delta {G}_{{{\text{CH}}}_{2}}$, a measure of solution polarity, which is similar to the strategy employed in molecular thermodynamic modeling of ${g}_{{\text{mic}}}$ (Nagarajan and Ruckenstein 1991; Müh et al. 2015; Bothe et al. 2023). Willauer et al. (2002) found a linear correlation between $\Delta {G}_{{{\text{CH}}}_{2}}$ and the difference in ${c}_{{\text{OE}}}$ between the two phases that is independent of the relative molecular weight (in this case, PEG1000, PEG2000, and PEG3400 were investigated). They also found a linear correlation between the logarithm of the distribution ratio and the number of carbon atoms in the alkyl chain. These findings support our analysis of the PEG-induced CMC shifts and provide a simple model for their understanding. Each OE unit in the aqueous phase changes the transfer free energy of the alkyl chain of the detergent in a way that the solubility of the monomer is increased and the micellization equilibrium shifted in favor of the monomers. The change of $\Delta {G}_{{{\text{CH}}}_{2}}$ in our case can be determined in the following way: From Eq. (57), we obtain the change of micellization free energy per detergent molecule in the micelle as

58 $$\frac{\mathrm{\Delta \Delta }{G}^{0}}{m}={k}_{{\text{B}}}T \frac{\kappa }{p} \frac{{c}_{{\text{OE}}}}{{c}_{{\text{tot}}}}$$

which is equivalent to $-{G}_{{\text{int}}}$ in Eq. (10) for ${N}_{1}=1$. The contribution of a methylene group is obtained from the change of $\kappa /p$ with the alkyl chain length $n$ multiplied by the thermal energy, which on the basis of Fig. 5 amounts to $3.2\ {k}_{{\text{B}}}T$. Thus, we have for the change of $\Delta {G}_{{{\text{CH}}}_{2}}$ due to PEG:

59 $$\mathrm{\Delta \Delta }{G}_{{{\text{CH}}}_{2}}=3.2\ {k}_{{\text{B}}}T \frac{{c}_{{\text{OE}}}}{{c}_{{\text{tot}}}}$$

**Fig. 6:**
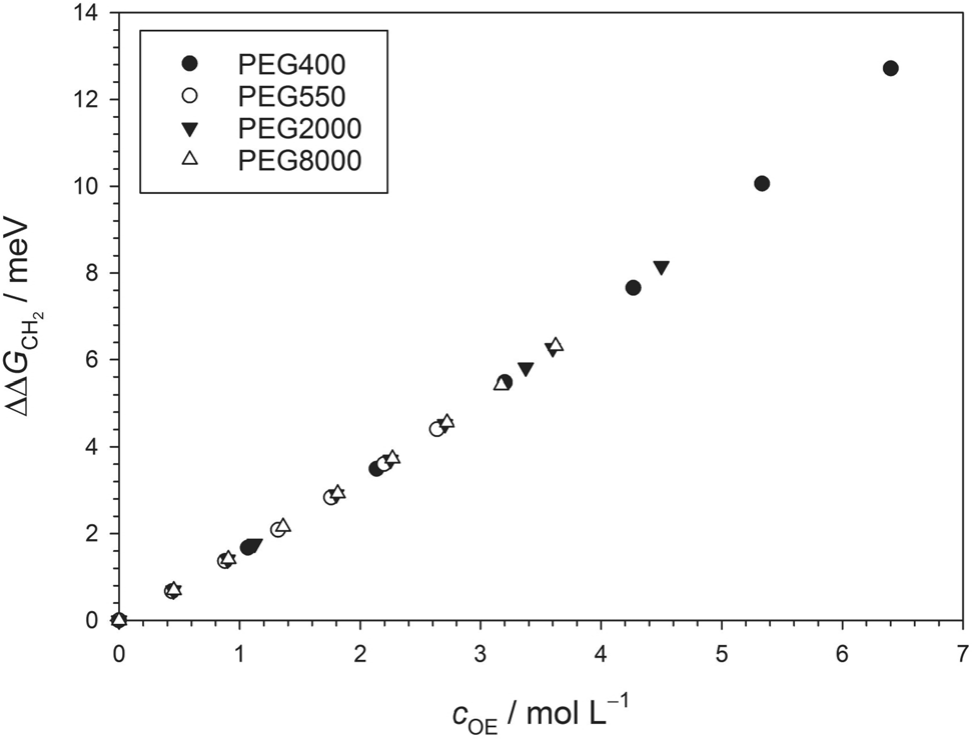
Dependence of the change of the transfer free energy of a methylene group, $\mathrm{\Delta \Delta }{G}_{{{\text{CH}}}_{2}}$, which is a measure of solution polarity, on the concentration of OE units, ${c}_{{\text{OE}}}$, in aqueous 100 mM PIPES (pH 7.0), 5 mM CaCl_2_ computed on the basis of Eq. (59) for the PEG concentrations of the DM data sets (cf. SI Table S1) and $T=298\ {\text{K}}$. Figure made with SigmaPlot 13 (© 2014 Systat Software Inc.)

Note that at constant temperature, $\mathrm{\Delta \Delta }{G}_{{{\text{CH}}}_{2}}$ depends linearly on the mole fraction of OE units, but due to the dependence of the total molarity on the amount of PEG (Eq. 51), it is a nonlinear function of ${c}_{{\text{OE}}}$ (Fig. 6). However, the nonlinearity is weak and a plot of ${c}_{{\text{tot}}}\mathrm{\Delta \Delta }{G}_{{{\text{CH}}}_{2}}$ versus ${c}_{{\text{OE}}}$ yields again a linear correlation (SI Fig. S7). The order of magnitude of $\mathrm{\Delta \Delta }{G}_{{{\text{CH}}}_{2}}$ for a change of ${c}_{{\text{OE}}}$ by 1 mol L^−1^ is about 0.2 kJ mol^−1^ at $T=298\ {\text{K}}$. This increment is about twice as large as the one found for the alcohol partitioning in the aqueous biphasic systems (Willauer et al. 2002), but in a similar range. The difference could be due the CMC shift being determined not solely by the alkyl chain of the detergent and/or an influence of other buffer components. Therefore, it would be of interest to characterize this measure of solution polarity in various buffer systems relevant for membrane protein crystallization and for different types of detergents.

Based on the simple idea that PEG enhances the solubility of detergent monomers, we can discuss other processes of interest in the context of membrane protein crystallization. One such process is the formation of a PDC characterized by the CSC. We note that a proper definition of the CSC in the spirit of the definition of the CMC (Bothe et al. 2023) still needs to be worked out. From a practical point of view, the CSC can be viewed as the minimal total detergent concentration that is needed to keep a given amount of membrane protein in a monodisperse solution in an aqueous phase (and hence it depends on the protein concentration). It is then expected that above the CSC, only PDCs with a complete detergent belt surrounding the hydrophobic (i.e., originally membrane-facing) protein parts are present. Below the CSC, there should be PDCs with an incomplete detergent belt. Based on our earlier work (Müh and Zouni 2008; Müh et al. 2015), we have suggested that the CSC should be larger (or at least not smaller) than the CMC and that the slot of detergent concentrations between CMC and CSC could be a suitable range for the formation of type-I membrane protein crystals that are detergent-depleted compared to type-II crystals (Michel 1983; Ostermeier and Michel 1997). On the other hand, type-II crystals should form above the CSC and most suitably close to the CSC, where the concentration of free micelles (i.e., not containing protein), which could perturb the crystallization process, is expected to be minimal. Thus, the CSC is considered by us to be an important quantity in the context of optimizing crystallization experiments. We note that the route to type-I crystals sketched above is hypothetical. In fact, type-I crystals are usually obtained in lipid cubic phase (LCP, also termed *in meso* (Birch et al. 2018; Kermani 2021)), but this is not applicable to PSII.

The formation of a PDC can be described by a mass action model similar to micelle formation (Müh and Zouni 2008). By ${{\text{P}}}_{1}$ we denote a protein monomer, that is the entity surrounded by $b$ detergent molecules in the PDC, ${{\text{P}}}_{1}{{\text{D}}}_{b}$. (In the case of PSII, ${{\text{P}}}_{1}$ is usually a dimeric core complex.) Without detergent, the protein monomer will likely form aggregates, which we assume here for simplicity to be monosdisperse with aggregation number $a$. Then the equilibrium for the solubilization of the protein is

60 $$ab {\text{D}}+{{\text{P}}}_{a} \rightleftharpoons a {{\text{P}}}_{1}{{\text{D}}}_{b}$$

The associated equilibrium constant can be written as

61 $${K}_{{\text{sol}}}=\frac{{X}_{{\text{PDC}}}^{a}}{{X}_{1}^{ab}{X}_{{{\text{P}}}_{a}}}={e}^{-\Delta {G}_{{\text{sol}}}^{0}/{k}_{{\text{B}}}T}$$

where “sol” stands for “solubilization” and ${X}_{1}$, ${X}_{{{\text{P}}}_{a}}$, and ${X}_{{\text{PDC}}}$ are, respectively, the mole fractions of detergent monomers, protein aggregates, and PDCs, while $\Delta {G}_{{\text{sol}}}^{0}$ is the standard free energy of solubilization. The latter can be decomposed on the basis of a thermodynamic cycle (Müh and Zouni 2008) according to

62 $$\Delta {G}_{{\text{sol}}}^{0}=\Delta {G}_{{\text{diss}}}^{0}+\Delta {G}_{{\text{belt}}}^{0}$$

Here, $\Delta {G}_{{\text{diss}}}^{0}$ and $\Delta {G}_{{\text{belt}}}^{0}$ are, respectively, the standard free energies of protein aggregate dissociation and detergent assembly into the belt. PEG is known to affect protein solubility and to induce effective interactions between proteins that are relevant for crystallization (Hitscherich et al. 2000; Kulkarni et al. 2000; Tanaka et al. 2003; Onuma et al. 2009). Therefore, it will certainly have an effect on $\Delta {G}_{{\text{diss}}}^{0}$, but this problem is beyond the scope of the present work. However, we can draw some conclusions about the effect of PEG on $\Delta {G}_{{\text{belt}}}^{0}$. Within the model that PEG affects the solubility of detergent monomers, it follows immediately that $\Delta {G}_{{\text{belt}}}^{0}$ is increased in a way similar to $\Delta {G}^{0}$ for micelle formation (see Eq. (57)), the only difference being the stoichiometric coefficient:

63 $$\mathrm{\Delta \Delta }{G}_{{\text{belt}}}^{0}={k}_{{\text{B}}}Tab \frac{\kappa }{p} \frac{{c}_{{\text{OE}}}}{{c}_{{\text{tot}}}}$$

Further, by analogy to Eq. (54), we can write a preliminary relation between ${K}_{{\text{sol}}}$ and the CSC:

64 $${\text{ln}}{K}_{{\text{sol}}}=-ab{\text{ln}}{X}_{{\text{CSC}}}$$

where ${X}_{{\text{CSC}}}={\text{CSC}}/{c}_{{\text{tot}}}$. We emphasize that Eq. (64) is at present a guess that requires further work to be substantiated. It is, however, reasonable to assume that PEG will shift the equilibrium in Eq. (60) towards the left side as it does with the micellization equilibrium in Eq. (17) and hence increase the CSC. Since it can be expected that $ab>m$, the shift of $\Delta {G}_{{\text{belt}}}^{0}$ will even be larger than that of $\Delta {G}^{0}$ for micelle formation. However, by analogy to Eq. (56), we hypothesize that

65 $${\text{ln}}\frac{{\text{CSC}}}{{c}_{{\text{tot}}}}- {\text{ln}}\frac{{\text{CSC}}\left(0\right)}{{c}_{{\text{tot}}}\left(0\right)}=\frac{\kappa }{p} \frac{{c}_{{\text{OE}}}}{{c}_{{\text{tot}}}}$$

so that the CSC shifts according to the CMC. We come to the conclusion that PEG plays a double role in membrane protein crystallization. It not only tunes the protein–protein interaction for crystal formation, but also shifts critical boundaries for detergent aggregation into micelles and PDCs.

What are the practical implications? The formation of type-II crystals is perturbed below the CSC because of protein aggregation and far above the CSC because of a higher concentration of free detergent micelles. Thus, there is a critical range of detergent concentrations. This range is shifted by PEG, so that it might be necessary to readjust the detergent concentration with regard to the PEG concentration. An experimental verification of such an optimization remains to be done. A difficulty is here the chemical quantification of detergent in the crystallization setup. From a theoretical point of view, the CSC remains to be properly defined and the concentration of free micelles as a function of total detergent concentration in the presence of PDCs remains to be computed on the basis of a thermodynamic model.

In the particular case of dimeric PSII, the PDC concentration in the crystallization setup is 30 µM (Kern et al. 2005; Hussein et al. 2018). According to our earlier analysis (Müh and Zouni 2005), this would require a minimum DDM concentration (CSC) of about 11 mM in the absence of PEG. However, the CSC determinations performed so far (Müh and Zouni 2005, 2008) were done at 100 times lower protein concentrations because of the applied optical measurement techniques. It remains unclear, whether the linear relationship between CSC and protein concentration holds for higher protein concentrations. The PEG concentration in a typical crystallization setup is in the order of 5–10% (w/v) PEG2000 (Hussein et al. 2018) corresponding to ${c}_{{\text{OE}}}=1.7\pm 0.6 \, {\text{mol}} \, {{\text{L}}}^{-1}$. According to the above estimates ($\kappa /p=17$ for $n=12$, ${c}_{{\text{tot}}}=51.15\pm 0.25 \, \mathrm{mol }\ { {\text{L}}}^{-1}$ according to Eq. (51) and applying Eq. (65)), this would increase the CSC for DDM to $18.5\pm 3.5 \, {\text{mM}}$. This value is in marked contrast to the nominal DDM concentration in the cyrstallization buffer of 0.29 mM. As mentioned above, a problem is that the real DDM concentration in the crystallization setup is actually not known. In the preparation, the PDCs have to be concentrated, which results in a yet undefined increase of the detergent concentration. If we assume that each PDC carries with it 360 DDM molecules (Müh and Zouni 2005), we arrive at an effective detergent conentration of ~ 11 mM. To reach 18.5 mM, the number $b$ would have to be around 600, which is probably too large. We see that there remain discrepancies that can only be resolved by determining the CSC at higher PDC concentrations and quantifying the detergent in the crystallization setup. Both problems are under study in our lab (Hart 2023; Kretzschmar 2023).

Besides direct crystallization, there is another route to type-I crystals. It has been demonstrated for dimeric PSII that dehydration procedures applied to type-II crystals can lead to a substantial detergent depletion resulting in a repacking to type-I crystals accompanied by a significant improvement in resolution (Hellmich et al. 2014; Kern et al. 2019). However, this trick works only with detergents of the oxyethylene type (C_12_E_8_) and not with alkyl maltosides. The polymer employed in the procedure, PEG5000 monomethyl ether (MME), was shown to increase the CMC of both C_12_E_8_ and DDM, albeit to a different extent with the CMC shift of the former detergent being slightly larger. These data show that the CMC shift is not solely determined by the alkyl chain, although they do not necessarily exclude that the alkyl chain is the dominating factor in the case of alkyl maltosides. Although other factors likely contribute to the detergent extraction such as the size and flexibility of the detergent head group, the solubility of detergent monomers in the solution outside the crystals is certainly important, and this is clearly enhanced by PEG. The fact that major progress in the structural biology of PSII has been made based on this type of detergent-depleted crystal (Ibrahim et al. 2015, 2020; Kern et al. 2018, 2019; Hussein et al. 2021; Bhowmick et al. 2023) demonstrates the importance of investigating how PEG affects the detergent behavior.

In summary, we have extended our earlier analysis of the effect of PEG2000 on the CMC of alkyl maltosides in a buffer suitable for the crystallization of PSII (Müh et al. 2015) to PEGs with different molecular weights and found that all PEGs behave similarly, if the effect is formulated in terms of the concentration of OE units. We also characterized the total molarity of PEG solutions in terms of OE units as this is needed for quantitative analyses. Finally, we linked our recent refined definition of the CMC (Bothe et al. 2023) to the analysis of the PEG effect. The underlying thermodynamic model was further developed, but requires extensions in future work in order to properly define the CSC and to capture micelle–micelle interactions as well as PEG-induced second virial coefficients, which are of prime interest for an understanding of membrane protein crystallization.

## Supplementary Information

Below is the link to the electronic supplementary material.

Supplementary file1 (PDF 1179 kb)

## Data Availability

All data are contained within the article or the SI.

## References

[CR1] Abuin EB, Lissi EA, Aspée A, Gonzalez FD, Varas JM (1997) Fluorescence of 8-anilinonaphthalene-1-sulfonate and properties of sodium dodecyl sulfate micelles in water-urea mixtures. J Colloid Interface Sci 186:332–338. 10.1006/jcis.1996.4648 9056362 10.1006/jcis.1996.4648

[CR2] Ago H, Kanaoka Y, Irikura D, Lam BK, Shimamura T, Austen KF, Miyano M (2007) Crystal structure of a human membrane protein involved in cysteinyl leukotriene biosynthesis. Nature 448:609–612. 10.1038/Nature05936 17632548 10.1038/nature05936

[CR3] Alpes H, Allmann K, Plattner H, Reichert J, Riek R, Schulz S (1986) Formation of large unilamellar vesicles using alkyl maltoside detergents. Biochim Biophys Acta Biomembr 862:294–302. 10.1016/0005-2736(86)90231-2

[CR4] Al-Soufi W, Pineiro L, Novo M (2012) A model for monomer and micellar concentrations in surfactant solutions: application to conductivity, NMR, diffusion, and surface tension data. J Colloid Interface Sci 370:102–110. 10.1016/j.jcis.2011.12.037 22265231 10.1016/j.jcis.2011.12.037

[CR5] Anthony O, Zana R (1994) Effect of temperature on the interactions between neutral polymers and a cationic and a nonionic surfactant in aqueous solutions. Langmuir 10:4048–4052. 10.1021/La00023a024

[CR6] Aoudia M, Zana R (1998) Aggregation behavior of sugar surfactants in aqueous solutions: effects of temperature and the addition of nonionic polymers. J Colloid Interface Sci 206:158–167. 10.1006/jcis.1998.5627 9761639 10.1006/jcis.1998.5627

[CR7] Aveyard R, Binks BP, Chen J, Esquena J, Fletcher PDI, Buscall R, Davies S (1998) Surface and colloid chemistry of systems containing pure sugar surfactant. Langmuir 14:4699–4709. 10.1021/La980519x

[CR8] Bhowmick A, Hussein R, Bogacz I, Simon PS, Ibrahim M, Chatterjee R, Doyle MD, Cheah MH, Fransson T, Chernev P, Kim IS, Makita H, Dasgupta M, Kaminsky CJ, Zhang M, Gaetcke J, Haupt S, Nangca II, Keable SM, Aydin AO, Tono K, Owada S, Gee LB, Fuller FD, Batyuk A, Alonso-Mori R, Holton JM, Paley DW, Moriarty NW, Mamedov F, Adams PD, Brewster AS, Dobbek H, Sauter NK, Bergmann U, Zouni A, Messinger J, Kern J, Yano J, Yachandra VK (2023) Structural evidence for intermediates during O_2_ formation in photosystem II. Nature 617:629–636. 10.1038/s41586-023-06038-z 37138085 10.1038/s41586-023-06038-z PMC10191843

[CR9] Birch J, Axford D, Foadi J, Meyer A, Eckhardt A, Thielmann Y, Moraes I (2018) The fine art of integral membrane protein crystallisation. Methods 147:150–162. 10.1016/j.ymeth.2018.05.014 29778646 10.1016/j.ymeth.2018.05.014

[CR10] Blankenship RE (2021) Molecular mechanisms of photosynthesis. Wiley

[CR11] Blouwolff J, Fraden S (2007) Crystallization conditions of membrane protein CLC-ec1: an example outside the crystallization slot. J Crystal Growth 303:546–553. 10.1016/j.jcrysgro.2007.01.028

[CR12] Bothe A, Zouni A, Müh F (2023) Refined definition of the critical micelle concentration and application to alkyl maltosides used in membrane protein research. RSC Adv 13:9387–9401. 10.1039/d2ra07440k 36968053 10.1039/d2ra07440k PMC10031436

[CR13] Broser M, Gabdulkhakov A, Kern J, Guskov A, Müh F, Saenger W, Zouni A (2010) Crystal structure of monomeric photosystem II from *Thermosynechococcus elongatus* at 3.6 Å resolution. J Biol Chem 285:26255–26262. 10.1074/jbc.M110.127589 20558739 10.1074/jbc.M110.127589 PMC2924040

[CR14] Cardona T, Sedoud A, Cox N, Rutherford AW (2012) Charge separation in photosystem II: a comparative and evolutionary overview. Biochim Biophys Acta Bioenerg 1817:26–43. 10.1016/j.bbabio.2011.07.012 10.1016/j.bbabio.2011.07.012 21835158

[CR15] Cecutti C, Focher B, Perly B, Zemb T (1991) Glycolipid self-assembly—micellar structure. Langmuir 7:2580–2585. 10.1021/La00059a031

[CR16] Chakraborty T, Ghosh S (2008) A unified survey of applicability of theories of mixed adsorbed film and mixed micellization. J Surfact Deterg 11:323–334. 10.1007/s11743-008-1095-1

[CR17] De Grip WJ, Bovee-Geurts PHM (1979) Synthesis and properties of alkylglucosides with mild detergent action: improved synthesis and purification of β-1-Octyl-, -nonyl-, and -decyl-glucose. Synthesis of β-1-Undecylglucose and β-1-dodecylmaltose. Chem Phys Lipids 23:321–335. 10.1016/0009-3084(79)90010-0

[CR18] De Vendittis E, Palumbo G, Parlato G, Bocchini V (1981) A fluorimetric method for the estimation of the critical micelle concentration of surfactants. Anal Biochem 115:278–286. 10.1016/0003-2697(81)90006-3 7304960 10.1016/0003-2697(81)90006-3

[CR19] de Wijn R, Melo DVM, Koua FHM, Mancuso AP (2022) Potential of time-resolved serial femtosecond crystallography using high repetition rate XFEL sources. Appl Sci 12:2551. 10.3390/app12052551

[CR20] Dey A, Banik R, Ghosh S (2021) Temperature comparative studies on self-assembly of sodium dodecyl sulphate and didodecyl dimethyl ammonium bromide in aqueous, brine, and trifluoroethanol media. J Surfact Deterg 24:459–472. 10.1002/jsde.12385

[CR21] Drummond CJ, Warr GG, Grieser F, Ninham BW, Evans DF (1985) Surface-properties and micellar interfacial microenvironment of *n*-dodecyl β-D-maltoside. J Phys Chem 89:2103–2109. 10.1021/J100256a060

[CR22] Dupuy C, Auvray X, Petipas C (1997) Anomeric effects on the structure of micelles of alkyl maltosides in water. Langmuir 13:3965–3967. 10.1021/La9604285

[CR23] Golub M, Hussein R, Ibrahim M, Hecht M, Wieland DCF, Martel A, Machado B, Zouni A, Pieper J (2020) Solution structure of the detergent-photosystem II core complex investigated by small-angle scattering techniques. J Phys Chem B 124:8583–8592. 10.1021/acs.jpcb.0c07169 32816484 10.1021/acs.jpcb.0c07169

[CR24] Golub M, Kölsch A, Feoktystov A, Zouni A, Pieper J (2021) Insights into solution structures of photosynthetic protein complexes from small-angle scattering methods. Crystals 11:203. 10.3390/Cryst11020203

[CR25] Golub M, Gatcke J, Subramanian S, Kölsch A, Darwish T, Howard JK, Feoktystov A, Matsarskaia O, Martel A, Porcar L, Zouni A, Pieper J (2022) “Invisible” detergents enable a reliable determination of solution structures of native photosystems by small-angle neutron scattering. J Phys Chem B 126:2824–2833. 10.1021/acs.jpcb.2c01591 35384657 10.1021/acs.jpcb.2c01591

[CR26] González-Tello P, Camacho F, Blázquez G (1994) Density and viscosity of concentrated aqueous solutions of polyethylene glycol. J Chem Eng Data 39:611–614. 10.1021/Je00015a050

[CR27] Hart O (2023) Quantification and control of C_12_E_8_ detergent concentration during isolation of photosystem 2. Master thesis, Humboldt Universität zu Berlin

[CR28] Hellmich J, Bommer M, Burkhardt A, Ibrahim M, Kern J, Meents A, Müh F, Dobbek H, Zouni A (2014) Native-like photosystem II superstructure at 2.44 Å resolution through detergent extraction from the protein crystal. Structure 22:1607–1615. 10.1016/j.str.2014.09.007 25438669 10.1016/j.str.2014.09.007

[CR29] Hitscherich C, Kaplan J, Allaman M, Wiencek J, Loll PJ (2000) Static light scattering studies of OmpF porin: implications for integral membrane protein crystallization. Protein Sci 9:1559–1566. 10.1110/ps.9.8.1559 10975577 10.1110/ps.9.8.1559 PMC2144733

[CR30] Hussein R, Ibrahim M, Chatterjee R, Coates L, Müh F, Yachandra VK, Yano J, Kern J, Dobbek H, Zouni A (2018) Optimizing crystal size of photosystem II by macroseeding: toward neutron protein crystallography. Cryst Growth Des 18:85–94. 10.1021/acs.cgd.7b00878 29962903 10.1021/acs.cgd.7b00878 PMC6020701

[CR31] Hussein R, Ibrahim M, Bhowmick A, Simon PS, Chatterjee R, Lassalle L, Doyle M, Bogacz I, Kim IS, Cheah MH, Gul S, de Lichtenberg C, Chernev P, Pham CC, Young ID, Carbajo S, Fuller FD, Alonso-Mori R, Batyuk A, Sutherlin KD, Brewster AS, Bolotovsky R, Mendez D, Holton JM, Moriarty NW, Adams PD, Bergmann U, Sauter NK, Dobbek H, Messinger J, Zouni A, Kern J, Yachandra VK, Yano J (2021) Structural dynamics in the water and proton channels of photosystem II during the S_2_ to S_3_ transition. Nat Commun. 10.1038/S41467-021-26781-Z 34764256 10.1038/s41467-021-26781-z PMC8585918

[CR32] Ibrahim M, Chatterjee R, Hellmich J, Tran R, Bommer M, Yachandra VK, Yano J, Kern J, Zouni A (2015) Improvements in serial femtosecond crystallography of photosystem II by optimizing crystal uniformity using microseeding procedures. Struct Dyn 2:041705. 10.1063/1.4919741 26726311 10.1063/1.4919741 PMC4697744

[CR33] Ibrahim M, Fransson T, Chatterjee R, Cheah MH, Hussein R, Lassalle L, Sutherlin KD, Young ID, Fuller FD, Gul S, Kim IS, Simon PS, de Lichtenberg C, Chernev P, Bogacz I, Pham CC, Orville AM, Saichek N, Northen T, Batyuk A, Carbajo S, Alonso-Mori R, Tono K, Owada S, Bhowmick A, Bolotovsky R, Mendez D, Moriarty NW, Holton JM, Dobbek H, Brewster AS, Adams PD, Sauter NK, Bergmann U, Zouni A, Messinger J, Kern J, Yachandra VK, Yano J (2020) Untangling the sequence of events during the S_2_ → S_3_ transition in photosystem II and implications for the water oxidation mechanism. Proc Natl Acad Sci USA 117:12624–12635. 10.1073/pnas.2000529117 32434915 10.1073/pnas.2000529117 PMC7293653

[CR34] Jordan P, Fromme P, Witt HT, Klukas O, Saenger W, Krauss N (2001) Three-dimensional structure of cyanobacterial photosystem I at 2.5 Å resolution. Nature 411:909–917. 10.1038/35082000 11418848 10.1038/35082000

[CR35] Jumpertz T, Tschapek B, Infed N, Smits SHJ, Ernst R, Schmitt L (2011) High-throughput evaluation of the critical micelle concentration of detergents. Anal Biochem 408:64–70. 10.1016/j.ab.2010.09.011 20850411 10.1016/j.ab.2010.09.011

[CR36] Junge W (2019) Oxygenic photosynthesis: history, status and perspective. Q Rev Biophys 52:e1. 10.1017/S0033583518000112 30670110 10.1017/S0033583518000112

[CR37] Kermani AA (2021) A guide to membrane protein X-ray crystallography. FEBS J 288:5788–5804. 10.1111/febs.15676 33340246 10.1111/febs.15676

[CR38] Kern J, Loll B, Lüneberg C, DiFiore D, Biesiadka J, Irrgang KD, Zouni A (2005) Purification, characterisation and crystallisation of photosystem II from *Thermosynechococcus elongatus* cultivated in a new type of photobioreactor. Biochim Biophys Acta Bioenerg 1706:147–157. 10.1016/j.bbabio.2004.10.007 10.1016/j.bbabio.2004.10.007 15620375

[CR39] Kern J, Chatterjee R, Young ID, Fuller FD, Lassalle L, Ibrahim M, Gul S, Fransson T, Brewster AS, Alonso-Mori R, Hussein R, Zhang M, Douthit L, de Lichtenberg C, Cheah MH, Shevela D, Wersig J, Seuffert I, Sokaras D, Pastor E, Weninger C, Kroll T, Sierra RG, Aller P, Butryn A, Orville AM, Liang MN, Batyuk A, Koglin JE, Carbajo S, Boutet S, Moriarty NW, Holton JM, Dobbek H, Adams PD, Bergmann U, Sauter NK, Zouni A, Messinger J, Yano J, Yachandra VK (2018) Structures of the intermediates of Kok’s photosynthetic water oxidation clock. Nature 563:421–425. 10.1038/s41586-018-0681-2 30405241 10.1038/s41586-018-0681-2 PMC6485242

[CR40] Kern J, Müh F, Zouni A (2019) Structural studies on tetrapyrrole containing proteins enabled by femtosecond X-ray pulses. In: Grimm B (ed) Metabolism, structure and function of plant tetrapyrroles: control mechanisms of chlorophyll biosynthesis and analysis of chlorophyll-binding proteins. Elsevier, London, pp 33–67

[CR41] King AD (1991) The solubility of gases in aqueous solutions of polyethylene glycols. J Colloid Interface Sci 144:579–585. 10.1016/0021-9797(91)90423-6

[CR42] Kretzschmar M (2023) Effect of the detergent C_12_E_8_ on the crystallization of tPSI and first XFel measurements. Master thesis, Humboldt Universität zu Berlin

[CR43] Kulkarni AM, Chatterjee AP, Schweizer KS, Zukoski CF (2000) Effects of polyethylene glycol on protein interactions. J Chem Phys 113:9863–9873. 10.1063/1.1321042

[CR44] Kunji ERS, Harding M, Butler PJG, Akamine P (2008) Determination of the molecular mass and dimensions of membrane proteins by size exclusion chromatography. Methods 46:62–72. 10.1016/j.ymeth.2008.10.020 18952172 10.1016/j.ymeth.2008.10.020

[CR45] Liljekvist P, Kronberg B (2000) Comparing decyl-β-maltoside and octaethyleneglycol mono *n*-decyl ether in mixed micelles with dodecyl benzenesulfonate: 1. Formation of mixed micelles. J Colloid Interface Sci 222:159–164. 10.1006/jcis.1999.6608 10662510 10.1006/jcis.1999.6608

[CR46] Lipfert J, Columbus L, Chu VB, Lesley SA, Doniach S (2007) Size and shape of detergent micelles determined by small-angle X-ray scattering. J Phys Chem B 111:12427–12438. 10.1021/Jp073016l 17924686 10.1021/jp073016l

[CR47] Liu CG, Desai KGH, Liu CS (2005) Solubility of valdecoxib in the presence of poly(ethylene glycol) 4000, poly(ethylene glycol) 6000, poly(ethylene glycol) 8000, and poly(ethylene glycol) 10 000 at (298.15, 303.15, and 308.15) K. J Chem Eng Data 50:278–282. 10.1021/je049667h

[CR48] Loll B, Kern J, Saenger W, Zouni A, Biesiadka J (2005) Towards complete cofactor arrangement in the 3.0 Å resolution structure of photosystem II. Nature 438:1040–1044. 10.1038/Nature04224 16355230 10.1038/nature04224

[CR49] Mazor Y, Borovikova A, Nelson N (2015) The structure of plant photosystem I super-complex at 2.8 Å resolution. eLife. 10.7554/eLife.07433 26076232 10.7554/eLife.07433 PMC4487076

[CR50] Michel H (1983) Crystallization of membrane-proteins. Trends Biochem Sci 8:56–59. 10.1016/0968-0004(83)90390-0

[CR51] Molina DM, Wetterholm A, Kohl A, McCarthy AA, Niegowski D, Ohlson E, Hammarberg T, Eshaghi S, Haeggstrom J, Nordlund PR (2007) Structural basis for synthesis of inflammatory mediators by human leukotriene C_4_ synthase. Nature 448:613–616. 10.1038/Nature06009 17632546 10.1038/nature06009

[CR52] Müh F, Zouni A (2005) Extinction coefficients and critical solubilisation concentrations of photosystems I and II from *Thermosynechococcus elongatus*. Biochim Biophys Acta Bioenerg 1708:219–228. 10.1016/j.bbabio.2005.03.005 10.1016/j.bbabio.2005.03.005 15953478

[CR53] Müh F, Zouni A (2008) Micelle formation in the presence of photosystem I. Biochim Biophys Acta Biomembr 1778:2298–2307. 10.1016/j.bbamem.2008.05.016 10.1016/j.bbamem.2008.05.016 18602888

[CR54] Müh F, Zouni A (2011) Light-induced water oxidation in photosystem II. Front Biosci 16:3072–3132. 10.2741/3900 10.2741/3900 21622223

[CR55] Müh F, Zouni A (2020) Structural basis of light-harvesting in the photosystem II core complex. Protein Sci 29:1090–1119. 10.1002/pro.3841 32067287 10.1002/pro.3841 PMC7184784

[CR56] Müh F, Glöckner C, Hellmich J, Zouni A (2012) Light-induced quinone reduction in photosystem II. Biochim Biophys Acta Bioenerg 1817:44–65. 10.1016/j.bbabio.2011.05.021 10.1016/j.bbabio.2011.05.021 21679684

[CR57] Müh F, DiFiore D, Zouni A (2015) The influence of poly(ethylene glycol) on the micelle formation of alkyl maltosides used in membrane protein crystallization. Phys Chem Chem Phys 17:11678–11691. 10.1039/c5cp00431d 25865704 10.1039/c5cp00431d

[CR58] Müller EA, Rasmussen P (1991) Densities and excess volumes in aqueous poly(ethylene glycol) solutions. J Chem Eng Data 36:214–217. 10.1021/Je00002a019

[CR59] Murakami S, Nakashima R, Yamashita E, Matsumoto T, Yamaguchi A (2006) Crystal structures of a multidrug transporter reveal a functionally rotating mechanism. Nature 443:173–179. 10.1038/Nature05076 16915237 10.1038/nature05076

[CR60] Murata T, Yamato I, Kakinuma Y, Leslie AGW, Walker JE (2005) Structure of the rotor of the V-type Na^+^-ATPase from *Enterococcus hirae*. Science 308:654–659. 10.1126/science.1110064 15802565 10.1126/science.1110064

[CR61] Nagarajan R, Ruckenstein E (1991) Theory of surfactant self-assembly - a predictive molecular thermodynamic approach. Langmuir 7:2934–2969. 10.1021/La00060a012

[CR62] Nelson N, Junge W (2015) Structure and energy transfer in photosystems of oxygenic photosynthesis. Annu Rev Biochem 84:659–683. 10.1146/annurev-biochem-092914-041942 25747397 10.1146/annurev-biochem-092914-041942

[CR63] Neutze R, Brändén G, Schertler GFX (2015) Membrane protein structural biology using X-ray free electron lasers. Curr Opin Struct Biol 33:115–125. 10.1016/j.sbi.2015.08.006 26342349 10.1016/j.sbi.2015.08.006

[CR64] Okubo T, Ise N (1969) Solubilities of naphthalene and biphenyl in aqueous polymer solutions. J Phys Chem 73:1488–2000. 10.1021/J100725a053

[CR65] Oliver RC, Lipfert J, Fox DA, Lo RH, Doniach S, Columbus L (2013) Dependence of micelle size and shape on detergent alkyl chain length and head group. PLoS One 8:e62488. 10.1371/journal.pone.0062488 23667481 10.1371/journal.pone.0062488 PMC3648574

[CR66] Onuma K, Furubayashi N, Shibata F, Kobayashi Y, Kaito S, Ohnishi Y, Inaka K (2009) Anomalous effect of poly(ethylene)glycol on intermolecular interaction and protein molecule association. Cryst Growth Des 9:2517–2524. 10.1021/cg900019e

[CR67] Orville AM (2020) Recent results in time resolved serial femtosecond crystallography at XFELs. Curr Opin Struct Biol 65:193–208. 10.1016/j.sbi.2020.08.011 33049498 10.1016/j.sbi.2020.08.011

[CR68] Ostermeier C, Michel H (1997) Crystallization of membrane proteins. Curr Opin Struct Biol 7:697–701. 10.1016/S0959-440x(97)80080-2 9345629 10.1016/s0959-440x(97)80080-2

[CR69] Özcan M (2022) Why equilibrium constants are unitless. J Phys Chem Lett 13:3507–3509. 10.1021/acs.jpclett.2c00314 35443779 10.1021/acs.jpclett.2c00314

[CR70] Palsdottir H, Lojero CG, Trumpower BL, Hunte C (2003) Structure of the yeast cytochrome *bc*_1_ complex with a hydroxyquinone anion Q_o_ site inhibitor bound. J Biol Chem 278:31303–31311. 10.1074/jbc.M302195200 12782631 10.1074/jbc.M302195200

[CR71] Phillips JN (1955) The energetics of micelle formation. Trans Faraday Soc 51:561–569. 10.1039/Tf9555100561

[CR72] Privé GG (2007) Detergents for the stabilization and crystallization of membrane proteins. Methods 41:388–397. 10.1016/j.ymeth.2007.01.007 17367711 10.1016/j.ymeth.2007.01.007

[CR73] Regupathi I, Govindarajan R, Amaresh SP, Murugesan T (2009) Densities and viscosities of polyethylene glycol 6000 + triammonium citrate + water systems. J Chem Eng Data 54:3291–3295. 10.1021/je9002898

[CR74] Rodrigues ML, Oliveira TF, Pereira IAC, Archer M (2006) X-ray structure of the membrane-bound cytochrome *c* quinol dehydrogenase NrfH reveals novel haem coordination. EMBO J 25:5951–5960. 10.1038/sj.emboj.7601439 17139260 10.1038/sj.emboj.7601439 PMC1698886

[CR75] Rosevear P, VanAken T, Baxter J, Ferguson-Miller S (1980) Alkyl glycoside detergents: a simpler synthesis and their effects on kinetic and physical properties of cytochrome c oxidase. Biochemistry 19:4108–4115. 10.1021/bi00558a032 6250583 10.1021/bi00558a032

[CR76] Santonicola MG, Yocum MA, Lenhoff AM, Kaler EW (2007) Self-assembly of medium-chain alkyl monoglucosides in ammonium sulfate solutions with poly(ethylene glycol). Langmuir 23:5358–5366. 10.1021/La063427d 17429988 10.1021/la063427d

[CR77] Shevela D, Kern JF, Govindjee G, Messinger J (2023) Solar energy conversion by photosystem II: principles and structures. Photosynth Res 156:279–307. 10.1007/s11120-022-00991-y 36826741 10.1007/s11120-022-00991-y PMC10203033

[CR78] Stroebel D, Choquet Y, Popot JL, Picot D (2003) An atypical haem in the cytochrome *b*_6_*f* complex. Nature 426:413–418. 10.1038/Nature02155 14647374 10.1038/nature02155

[CR79] Stubenrauch C (2001) Sugar surfactants—aggregation, interfacial, and adsorption phenomena. Curr Opin Colloid Interface Sci 6:160–170. 10.1016/S1359-0294(01)00080-2

[CR80] Tanaka S, Ataka M, Onuma K, Kubota T (2003) Rationalization of membrane protein crystallization with polyethylene glycol using a simple depletion model. Biophys J 84:3299–3306. 10.1016/S0006-3495(03)70054-X 12719259 10.1016/S0006-3495(03)70054-X PMC1302890

[CR81] Thonghin N, Kargas V, Clews J, Ford RC (2018) Cryo-electron microscopy of membrane proteins. Methods 147:176–186. 10.1016/j.ymeth.2018.04.018 29702228 10.1016/j.ymeth.2018.04.018

[CR82] Tsamaloukas AD, Beck A, Heerklotz H (2009) Modeling the micellization behavior of mixed and pure *n*-alkyl-maltosides. Langmuir 25:4393–4401. 10.1021/la8033935 19366219 10.1021/la8033935

[CR83] Tummino PJ, Gafni A (1993) Determination of the aggregation number of detergent micelles using steady-state fluorescence quenching. Biophys J 64:1580–1587. 10.1016/S0006-3495(93)81528-5 8324192 10.1016/S0006-3495(93)81528-5 PMC1262485

[CR84] Umena Y, Kawakami K, Shen JR, Kamiya N (2011) Crystal structure of oxygen-evolving photosystem II at a resolution of 1.9 Å. Nature 473:55–60. 10.1038/nature09913 21499260 10.1038/nature09913

[CR85] Van den Mooter G, Augustijns P, Kinget R (1998) Application of the thermodynamics of mobile order and disorder to explain the solubility of temazepam in aqueous solutions of polyethylene glycol 6000. Int J Pharm 164:81–89. 10.1016/S0378-5173(97)00400-6

[CR86] Warr GG, Drummond CJ, Grieser F, Ninham BW, Evans DF (1986) Aqueous-solution properties of nonionic *n*-dodecyl β-D-maltoside micelles. J Phys Chem 90:4581–4586. 10.1021/J100410a022

[CR87] Wennerström H, Lindman B (1979) Micelles. Physical chemistry of surfactant association. Phys Rep 52:1–86. 10.1016/0370-1573(79)90087-5

[CR88] Willauer HD, Huddleston JG, Rogers RD (2002) Solvent properties of aqueous biphasic systems composed of polyethylene glycol and salt characterized by the free energy of transfer of a methylene group between the phases and by a linear solvation energy relationship. Ind Eng Chem Res 41:2591–2601. 10.1021/ie0107800

[CR89] Yano J, Yachandra V (2014) Mn_4_Ca cluster in photosynthesis: where and how water is oxidized to dioxygen. Chem Rev 114:4175–4205. 10.1021/cr4004874 24684576 10.1021/cr4004874 PMC4002066

[CR90] Yernool D, Boudker O, Jin Y, Gouaux E (2004) Structure of a glutamate transporter homologue from *Pyrococcus horikoshii*. Nature 431:811–818. 10.1038/Nature03018 15483603 10.1038/nature03018

[CR91] Zafarani-Moattar MT, Salabat A, Kabiribadr M (1995) Volumetric properties of PEG + salt + water. J Chem Eng Data 40:559–562. 10.1021/Je00019a003

[CR92] Zhang L, Somasundaran P, Maltesh C (1996) Electrolyte effects on the surface tension and micellization of *n*-dodecyl β-D-maltoside solutions. Langmuir 12:2371–2373. 10.1021/La950670w

