# Supplementary material for: Towards understanding the crystallization of photosystem II: influence of poly(ethylene glycol) of various molecular sizes on the micelle formation of alkyl maltosides
Source: Photosynth Res. 2024 Mar 15;162(2-3):273–89. doi: 10.1007/s11120-024-01079-5 (PMC11615006; doi:10.1007/s11120-024-01079-5)
Supplement: Supplementary file 1 — Supplementary file1 (PDF 1179 kb) [file 11120_2024_1079_MOESM1_ESM.pdf]

Supplementary Information

Towards understanding the crystallization of photosystem II: Influence of poly(ethylene glycol) of various molecular sizes on the micelle formation of alkyl maltosides

Frank Müh,[1] Adrian Bothe,[2] Athina Zouni[3]

[1] Institut für Theoretische Physik, Johannes Kepler Universität Linz, Altenberger Strasse 69, A-4040 Linz, Austria.

[2] Institut für Molekularbiologie und Biophysik, ETH Zürich, HPK, Otto-Stern-Weg 5, CH-8093 Zürich, Switzerland.

[3] Institut für Biologie, Humboldt Universität zu Berlin, Leonor-Michaelis-Haus, Philippstrasse 13, D-10095 Berlin, Germany.

Corresponding author: Frank Müh, frank.mueh@jku.at

Content

Tables S1 – S3

Figures S1 – S8

Derivation and Discussion of Eq. (36)

Derivation and Discussion of Eq. (51)

Draft model including micelle-PEG interactions and molecular volume effects

Table S1: Original CMC values in molarity units (mM) obtained from graphical extrapolation of ANS titration curves.

M_r	% (w/v) PEG	CMC / mmol L^{-1}		
		DM	UDM	DDM
400	0	1.300*	0.380*	0.090*
	5	1.410	0.410	0.143
	10	1.770	0.495	0.207
	15	2.210	0.540	0.260
	20	3.030	0.685	0.430
	25	4.100	0.790	0.570
	30	5.200	–	–
550	0	1.300*	0.380*	0.090*
	2	1.400	0.390	0.094
	4	1.420	0.480	0.132
	8	1.650	0.570	0.132
	12	1.700	0.730	0.190
2000	0	1.300*	0.380*, 0.390	0.090*
	2	–	0.400*, 0.480	0.095*
	4	1.400*	0.450*, 0.570	0.110*
	5	1.290	0.430	0.147
	6	–	0.490*, 0.560	0.120*
	7.5	–	0.460	–
	8	1.750*	0.550*, 0.710	0.140*
	10	1.850	0.600*, 0.490, 0.740	0.140*, 0.224
	12	2.000*	0.650*, 0.740	0.180*
	12.5	–	0.805	–
	14	–	0.700*, 0.770	–
	15	2.640	0.880	0.305
	16	2.400*	0.750*, 0.900	0.180*
	20	3.300	–	–
8000	0	1.300*	0.380*	0.090*
	2	1.400		–
	4	1.600	0.480	0.130
	6	2.000		–
	8	–	0.560	0.190
	12	2.350	0.760	0.210
	16	2.400	–	–

* Published before: Müh F, DiFiore D and Zouni A (2015) The influence of poly(ethylene glycol) on the micelle formation of alkyl maltosides used in membrane protein crystallization. Phys Chem Chem Phys 17: 11678-11691.

Table S2 Results of linear regressions based on Eq. (52) performed separately for each PEG type. The fitted lines can be seen in Fig. S1-S3.

M_r	DM		UDM		DDM	
	slope	R^2	slope	R^2	slope	R^2
400	10.7 ± 0.3	0.9879	7.8 ± 0.2	0.9907	18.1 ± 0.7	0.9730
550	7.6 ± 0.5	0.9553	14.0 ± 0.7	0.9809	15.6 ± 1.6	0.9119
2000	10.5 ± 0.6	0.9337	12.7 ± 0.6	0.8250	15.4 ± 1.4	0.7758
8000	11.6 ± 1.1	0.8805	14.1 ± 0.5	0.9905	19.3 ± 1.7	0.9343

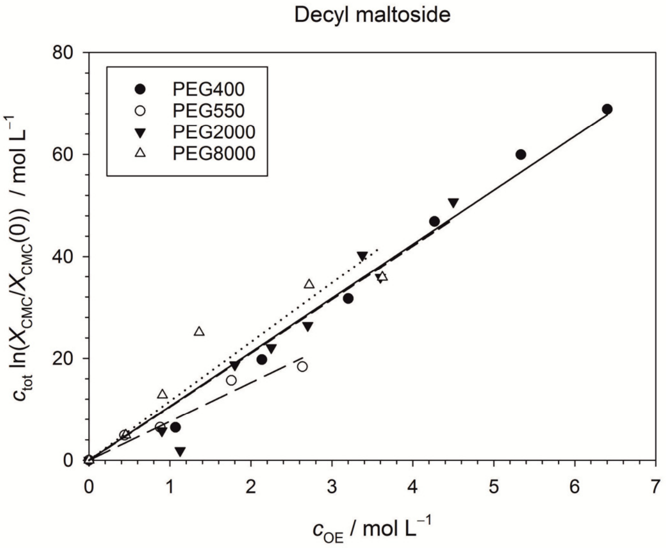

Fig. S1 Plots of $c_{\text{tot}}\ \ln(X_{\text{CMC}}(c_{\text{OE}})/X_{\text{CMC}}(0))$ (left side of Eq. (52)) versus c_{OE} for DM and the indicated PEG types. The slope of the plot is κ/p. The solid lines are linear regressions performed separately for each PEG type with zero intercept and the slopes given in Table S2: PEG400 (solid line), PEG550 (long dashed line), PEG2000 (short dashed line), PEG8000 (dotted line). Note that the lines for PEG400 and PEG2000 are on top of each other. Figure and fits made with SigmaPlot 13 (© 2014 Systat Software Inc.)

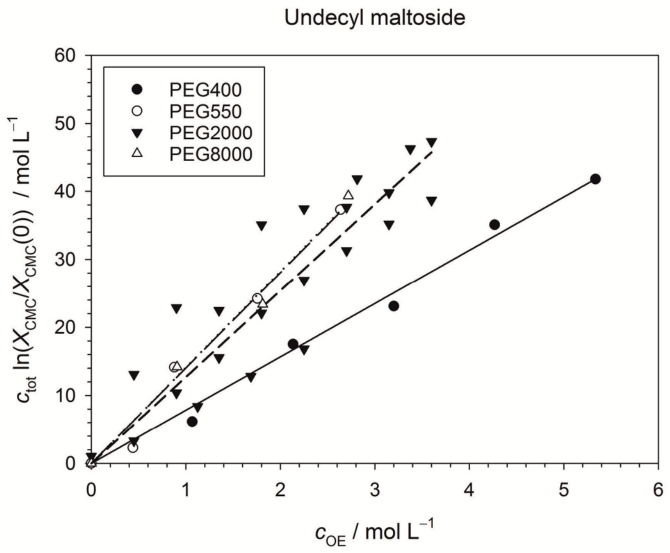

Fig. S2 Plots of $c_{\text{tot}}\ \ln(X_{\text{CMC}}(c_{\text{OE}})/X_{\text{CMC}}(0))$ (left side of Eq. (52)) versus c_{OE} for UDM and the indicated PEG types. The slope of the plot is κ/p. The solid lines are linear regressions performed separately for each PEG type with zero intercept and the slopes given in Table S2: PEG400 (solid line), PEG550 (long dashed line), PEG2000 (short dashed line), PEG8000 (dotted line). Note that the lines for PEG550 and PEG8000 are on top of each other. Figure and fits made with SigmaPlot 13 (© 2014 Systat Software Inc.)

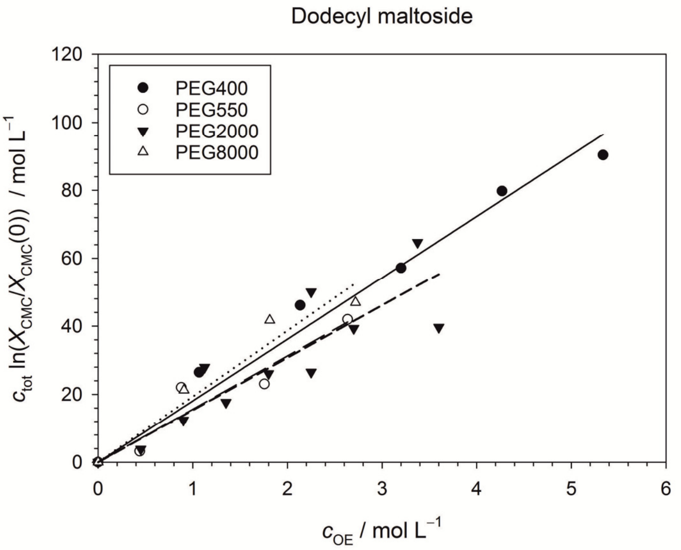

Fig. S3 Plots of $c_{\text{tot}}\ \ln(X_{\text{CMC}}(c_{\text{OE}})/X_{\text{CMC}}(0))$ (left side of Eq. (52)) versus c_{OE} for DDM and the indicated PEG types. The slope of the plot is κ/p. The solid lines are linear regressions performed separately for each PEG type with zero intercept and the slopes given in Table S2: PEG400 (solid line), PEG550 (long dashed line), PEG2000 (short dashed line), PEG8000 (dotted line). Note that the lines for PEG550 and PEG2000 are on top of each other. Figure and fits made with SigmaPlot 13 (© 2014 Systat Software Inc.)

Table S3 Results of linear regressions based on plots of ln(CMC/CMC(0)) versus χ performed separately for each PEG type. The fitted lines can be seen in Fig. S4-S6.

M_r	DM		UDM		DDM	
	slope	R^2	slope	R^2	slope	R^2
400	0.0434 ± 0.0021	0.9645	0.0279 ± 0.0013	0.9732	0.0756 ± 0.0018	0.9901
550	0.0247 ± 0.0019	0.9395	0.0529 ± 0.0029	0.9749	0.0599 ± 0.0063	0.9012
2000	0.0410 ± 0.0029	0.9025	0.0501 ± 0.0026	0.7987	0.0617 ± 0.0059	0.7466
8000	0.0449 ± 0.0042	0.8821	0.0552 ± 0.0024	0.9842	0.0786 ± 0.0062	0.9419

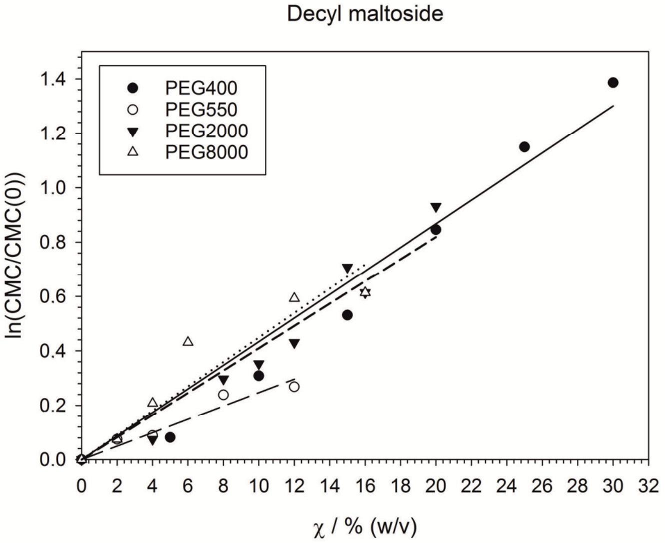

Fig. S4 Plots of ln(CMC/CMC(0)) versus χ for DM and the indicated PEG types. The solid lines are linear regressions performed separately for each PEG type with zero intercept and the slopes given in Table S3: PEG400 (solid line), PEG550 (long dashed line), PEG2000 (short dashed line), PEG8000 (dotted line). Figure and fits made with SigmaPlot 13 (© 2014 Systat Software Inc.)

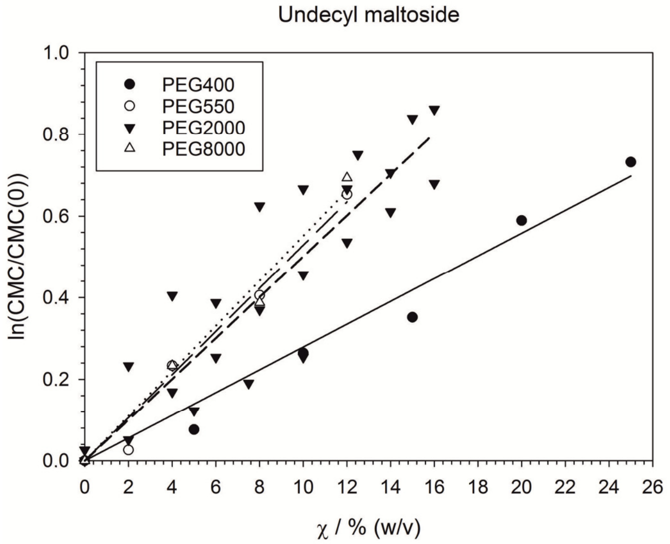

Fig. S5 Plots of ln(CMC/CMC(0)) versus χ for UDM and the indicated PEG types. The solid lines are linear regressions performed separately for each PEG type with zero intercept and the slopes given in Table S3: PEG400 (solid line), PEG550 (long dashed line), PEG2000 (short dashed line), PEG8000 (dotted line). Figure and fits made with SigmaPlot 13 (© 2014 Systat Software Inc.)

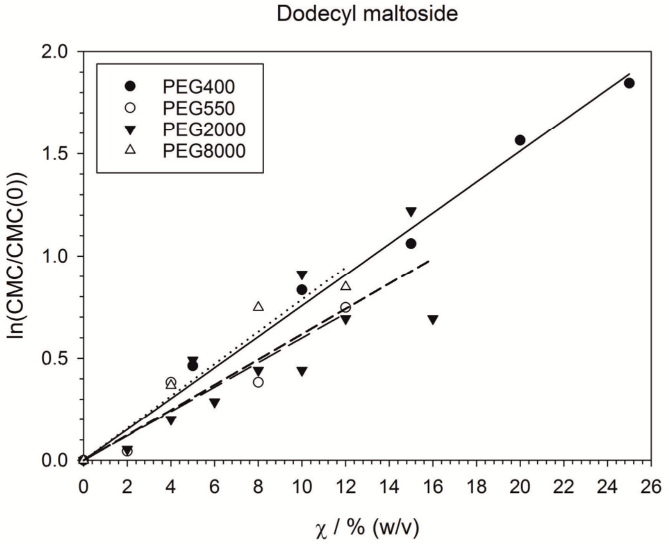

Fig. S6 Plots of ln(CMC/CMC(0)) versus χ for DDM and the indicated PEG types. The solid lines are linear regressions performed separately for each PEG type with zero intercept and the slopes given in Table S3: PEG400 (solid line), PEG550 (long dashed line), PEG2000 (short dashed line), PEG8000 (dotted line). Figure and fits made with SigmaPlot 13 (© 2014 Systat Software Inc.)

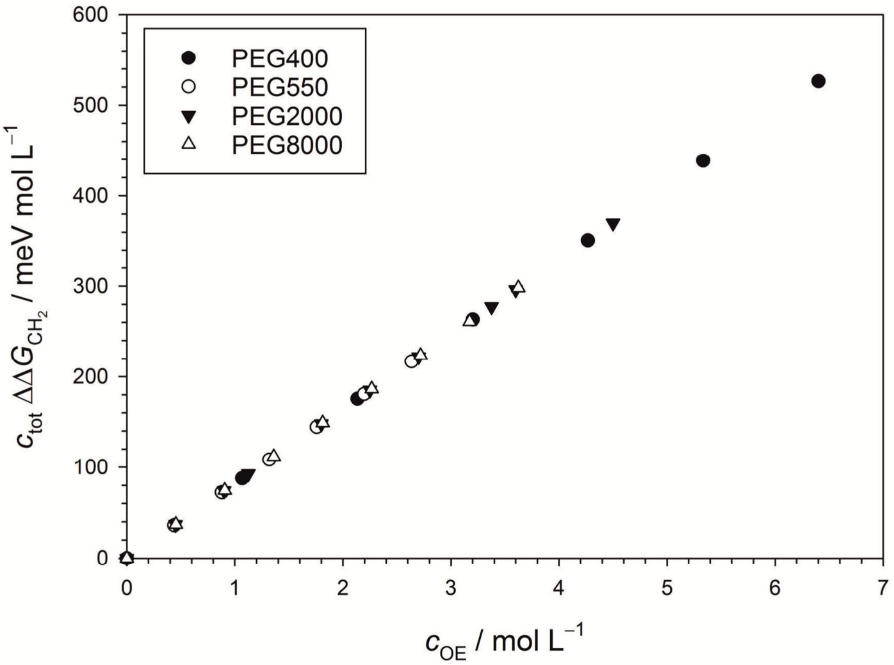

Fig. S7 Dependence of $c_{tot}\Delta\Delta G_{CH_2}$ on the concentration of OE-units, c_{OE}, in aqueous 100 mM PIPES (pH 7.0), 5 mM $CaCl_2$ computed on the basis of Eq. (59) for the PEG concentrations of the DM data sets (cf. SI Table S1) and T = 298 K. Figure made with SigmaPlot 13 (© 2014 Systat Software Inc.)

Derivation and Discussion of Eq. (36)

Eq. (36) is obtained by plugging Eq. (35) into Eq. (21) and using the limit $m \to \infty$. One may wonder whether it is justified to use Eq. (35) as an approximation for y in this context. To see this, we first estimate the order of magnitude of the terms occurring in Eq. (27). This estimate is based on the results of applying Eq. (36), so that it is rather a consistency check. The terms we want to evaluate contain JX_{P}, whose magnitude is given by

$$|JX_{\mathrm{P}}| = \frac{\kappa}{p} p \frac{c_{\mathrm{P}}}{c_{\mathrm{tot}}} = \frac{\kappa}{p} \frac{c_{\mathrm{OE}}}{c_{\mathrm{tot}}} = \frac{\kappa}{p} \frac{1}{c_{\mathrm{tot}}} \frac{10\,p}{M_{\mathrm{P}}} \chi < \frac{\kappa}{p} \frac{0.23\,\chi\,\mathrm{mol\,g^{-1}}}{c_{\mathrm{tot}}} \tag{S1}$$

To estimate an upper limit, we note that we have $\kappa/p < 20$ from the fits, and the experiments are done for $\chi < 50$ % (w/v). For these values, we obtain $|JX_{\mathrm{P}}| = 7.5$. As an upper limit for y, we use $y = 2 \times 10^{-4}$ for DM as well as $y = 2 \times 10^{-5}$ for UDM and DDM based on the highest CMC values (using the fact that $x \approx y$ around the CMC). Using these data and $m = 85, 106, 140$ (Bothe et al. 2023), one can see that all terms to higher order in y in Eq. (27) can be neglected compared to terms linear in y or not containing y, which is true essentially because $y \ll 1$. Then, we have the approximation

$$x = y + \frac{m(m-2)y}{m^2(2m-1) + b_1 y} \quad \Longrightarrow \quad x = \frac{m^2(2m-1)y + b_1 y^2 + m(m-2)y}{m^2(2m-1) + b_1 y} \tag{S2}$$

Neglecting again terms quadratic in y, we obtain

$$x = \frac{m^2(2m-1)y + m(m-2)y}{m^2(2m-1) + b_1 y}$$

$$\Longrightarrow \quad m^2(2m-1)x + b_1 xy = m^2(2m-1)y + m(m-2)y \tag{S3}$$

Since x is in the same order of magnitude as y around the CMC, we can also neglect terms containing xy, so that b_1 drops out and we obtain Eq. (35). Plugging it into Eq. (21) yields

$$g_{\mathrm{mic}} = \frac{(m-1)}{m} \ln X_{\mathrm{CMC}} + \frac{1}{m} \ln\left\{\frac{m(2m^2-m)^m}{(m-2)(2m^2-2)^{m-1}}\right\} - JX_{\mathrm{P}}\left[X_{\mathrm{CMC}} \frac{(m-1)(2m^2-m)}{m(2m^2-2)} - 1\right] \tag{S4}$$

which for $J = 0$ coincides with the result found by Bothe et al. (2023). For $J \neq 0$, it contains an additional term. Eq. (36) is obtained in the limit $m \to \infty$ (with the definition of κ in Eq. (37)). Bothe et al. (2023) found this limit to be appropriate for DM, UDM, and DDM, and it is used here to facilitate the analysis. To further substantiate it, we show in Fig. S8 plots of the various functions of m occurring in Eq. (S4). It can be seen that the factor multiplying X_{CMC} in the last term on the right hand side of Eq. (S4) approaches unity as fast as the prefactor of $\ln X_{\mathrm{CMC}}$, so that both factors are of minor importance for aggregation numbers around 100. The logarithmic factor, which for $m \approx 100$ is in the order of 0.1 and not necessarily negligible, drops out in the computation of $\ln(X_{\mathrm{CMC}}/X_{\mathrm{CMC}}(0))$. Therefore, Eq. (36) is sufficient for an analysis of the PEG-induced CMC shift and still compatible with the refined definition of the CMC based on Eq. (22). Note, however, that we assume that m is not affected by PEG.

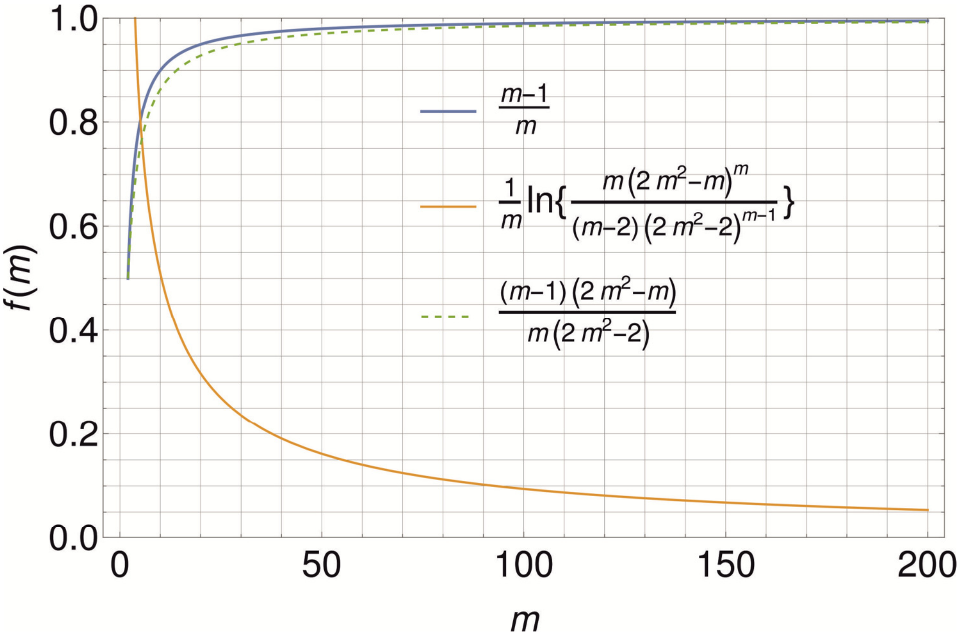

Fig. S8 Illustration of the various functions of m occurring in Eq. (S4). Plot and Figure made with Mathematica 13.0.1.0 (© 1988−2022 Wolfram Research)

Derivation and Discussion of Eq. (51)

We start by writing Eq. (48) in units of g dm^{-3} (at the risk of stating the obvious, we note that $\mathrm{L} = \mathrm{dm}^3$):

$$\rho = 1013.5\ \mathrm{g\,dm^{-3}} + 6.7\ \frac{\mathrm{g\,dm^{-3}}}{\mathrm{mol\,L^{-1}}}\ c_{\mathrm{OE}} \tag{S5}$$

Then, we plug Eq. (44) into Eq. (45):

$$c_{\mathrm{tot}} = \frac{\rho}{M_{\mathrm{wat}}} + c_{\mathrm{P}}\left(1 - \frac{M_{\mathrm{P}}}{M_{\mathrm{wat}}}\right) + k_{\cos} = \frac{\rho}{M_{\mathrm{wat}}} + \frac{c_{\mathrm{OE}}}{p}\left(1 - \frac{M_{\mathrm{P}}}{M_{\mathrm{wat}}}\right) + k_{\cos} \tag{S6}$$

Using Eq. (41) and the fact that $M_{\mathrm{wat}} = M_{\mathrm{H_2O}}$, we obtain

$$c_{\mathrm{tot}} = \frac{\rho}{M_{\mathrm{wat}}} + c_{\mathrm{OE}} \frac{\left(1 - \frac{M_{\mathrm{P}}}{M_{\mathrm{wat}}}\right)}{\left(\frac{M_{\mathrm{P}} - M_{\mathrm{H_2O}}}{M_{\mathrm{OE}}}\right)} + k_{\cos} = \frac{\rho}{M_{\mathrm{wat}}} + c_{\mathrm{OE}} \frac{M_{\mathrm{OE}}}{M_{\mathrm{H_2O}}} \frac{\left(1 - \frac{M_{\mathrm{P}}}{M_{\mathrm{wat}}}\right)}{\left(\frac{M_{\mathrm{P}}}{M_{\mathrm{H_2O}}} - 1\right)} + k_{\cos}$$

$$= \frac{\rho}{M_{\mathrm{wat}}} - c_{\mathrm{OE}} \frac{M_{\mathrm{OE}}}{M_{\mathrm{wat}}} \frac{\left(1 - \frac{M_{\mathrm{P}}}{M_{\mathrm{wat}}}\right)}{\left(1 - \frac{M_{\mathrm{P}}}{M_{\mathrm{wat}}}\right)} + k_{\cos} = \frac{\rho}{M_{\mathrm{wat}}} - c_{\mathrm{OE}} \frac{M_{\mathrm{OE}}}{M_{\mathrm{wat}}} + k_{\cos} \tag{S7}$$

Plugging Eq. (S5) and the numerical values for the molar masses and $k_{\cos}$ into Eq. (S7), we finally obtain

$$c_{\mathrm{tot}} = \frac{1013.5\ \mathrm{g\,dm^{-3}} + 6.7\ \frac{\mathrm{g\,dm^{-3}}}{\mathrm{mol\,L^{-1}}}\ c_{\mathrm{OE}}}{18.015\ \mathrm{g\,mol^{-1}}} - c_{\mathrm{OE}} \frac{44.053\ \mathrm{g\,mol^{-1}}}{18.015\ \mathrm{g\,mol^{-1}}} - 1.5935 \frac{\mathrm{mol}}{\mathrm{L}}$$

$$= 54.665 \frac{\mathrm{mol}}{\mathrm{L}} - \left(\frac{6.7 - 44.053}{18.015}\right) c_{\mathrm{OE}} = 54.665 \frac{\mathrm{mol}}{\mathrm{L}} - 2.073 c_{\mathrm{OE}} \tag{S8}$$

The somewhat counterintuitive result, that the number of moles for a given concentration of OE units is independent of the actual number of OE units in the polymer chain p, can be rationalized as follows: Let us assume for simplicity that we have only water and PEG. Let us further assume that one OE unit displaces q water molecules from the solution and the end groups of PEG (H-…-OH) displace one water molecule. Then, $qp + 1$ moles are removed per one mole of PEG and one mole (i. e., the PEG chain itself) is added. Thus, if c_{wat} is the molarity of water, we have for the PEG solution

$$c_{\mathrm{tot}} = c_{\mathrm{wat}} - (qp + 1)c_{\mathrm{P}} + c_{\mathrm{P}} = c_{\mathrm{wat}} - qpc_{\mathrm{P}} = c_{\mathrm{wat}} - qc_{\mathrm{OE}} \tag{S9}$$

The assumption that the end groups displace one water molecule may be supported by the observation that the ratio of end groups to OE units has no measurable influence on the density. For our buffer, we found $q = 2.073$. This result indicates that an OE unit displaces about two water molecules, which have a smaller relative molecular weight (36.03) than one OE unit (44.053) in accordance with the increase of the density of the PEG solution with PEG concentration. More precisely, $44.053 - 2.073 \times 18.015 = 6.708$ corresponds to the slope in Eq. (S5).

Draft model including micelle-PEG interactions and molecular volume effects

In the draft model, G_{mix} and G_{int} occurring in Eq. (1) of the main text are modified so that excluded volume effects are considered in G_{mix} and the interaction of PEG with all types of detergent aggregates is taken into account in G_{int}.

The basic idea of considering excluded volume effects in the entropy of mixing as formulated by Hildebrand[1] has been described by Bothe at al.[2] For a two-component system, the entropy of mixing is obtained by considering for each component the expansion from its free volume $N_i(\mathrm{v}_i - b_i)$ in the pure state to its free volume $V - N_1 b_1 - N_2 b_2$ in the mixture, where V is the actual volume of the solution:

$$\Delta S = k_{\mathrm{B}}\left[N_1 \ln\left\{\frac{V - N_1 b_1 - N_2 b_2}{N_1(\mathrm{v}_1 - b_1)}\right\} + N_2 \ln\left\{\frac{V - N_1 b_1 - N_2 b_2}{N_2(\mathrm{v}_2 - b_2)}\right\}\right] \quad \text{(S10)}$$

Here, N_i and v_i are, respectively, the particle number and volume occupied by one particle of species i. A simplifying assumption is that the solution is additive, i. e., there is no volume change due to mixing:

$$V = N_1 \mathrm{v}_1 + N_2 \mathrm{v}_2 \quad \text{(S11)}$$

Then, Eq. (S10) becomes

$$\frac{\Delta S}{k_{\mathrm{B}}} = N_1 \ln\left\{\frac{N_1(\mathrm{v}_1 - b_1) + N_2(\mathrm{v}_2 - b_2)}{N_1(\mathrm{v}_1 - b_1)}\right\} + N_2 \ln\left\{\frac{N_1(\mathrm{v}_1 - b_1) + N_2(\mathrm{v}_2 - b_2)}{N_2(\mathrm{v}_2 - b_2)}\right\} \quad \text{(S12)}$$

With these settings, we now consider an aqueous solution (index "w" for water) containing besides detergent aggregates with index ν (including the case $\nu = 1$ for detergent monomers) also PEG with index P and other co-solutes with index α. We introduce the abbreviations

$$\omega_i = \mathrm{v}_i - b_i \quad \text{(S13)}$$

and

$$\varpi = N_{\mathrm{w}}\omega_{\mathrm{w}} + N_{\mathrm{P}}\omega_{\mathrm{P}} + \sum_{\nu} N_{\nu}\omega_{\nu} + \sum_{\alpha} N_{\alpha}\omega_{\alpha} \quad \text{(S14)}$$

so that the free energy of mixing becomes

$$G_{\text{mix}} = -\beta^{-1}\left[N_{\mathrm{w}} \ln\left\{\frac{\varpi}{N_{\mathrm{w}}\omega_{\mathrm{w}}}\right\} + N_{\mathrm{P}} \ln\left\{\frac{\varpi}{N_{\mathrm{P}}\omega_{\mathrm{P}}}\right\} + \sum_{\nu} N_{\nu} \ln\left\{\frac{\varpi}{N_{\nu}\omega_{\nu}}\right\} + \sum_{\alpha} N_{\alpha} \ln\left\{\frac{\varpi}{N_{\alpha}\omega_{\alpha}}\right\}\right] \quad \text{(S15)}$$

To model G_{int}, we employ the Bragg-Williams approximation:[3]

---

[1] Hildebrand JH (1947) The Entropy of Solution of Molecules of Different Size. J Chem Phys 15: 225-228. https://doi.org/10.1063/1.1746484.

[2] Bothe A, Zouni A and Müh F (2023) Refined definition of the critical micelle concentration and application to alkyl maltosides used in membrane protein research. RSC Adv 13: 9387-9401. https://doi.org/10.1039/d2ra07440k.

[3] See: (a) Dill KA and Bromberg S (2011) Molecular Driving Forces. Garland Science, Taylor & Francis, New York. (b) Hill TL (1960, 1986) An Introduction to Statistical Thermodynamics. Dover, New York.

$$G_{\mathrm{int}} = \beta^{-1} \sum_{\nu} J_\nu \frac{N_{\mathrm{P}} N_\nu}{\bar{N}} \tag{S16}$$

Here, we consider for simplicity only interactions between PEG molecules and detergent aggregates. The coupling constant J_ν for $\nu = 1$ correspond to J in Eq. (10) of the main text.

Eq. (12) of the main text requires the computation of the partial derivative

$$\frac{\partial G}{\partial N_\nu} = \frac{\partial G_{\mathrm{f}}}{\partial N_\nu} + \frac{\partial G_{\mathrm{mix}}}{\partial N_\nu} + \frac{\partial G_{\mathrm{int}}}{\partial N_\nu} \tag{S17}$$

We have simply

$$\frac{\partial G_{\mathrm{f}}}{\partial N_\nu} = \nu \mu_\nu^{\mathrm{o}} \tag{S18}$$

For the derivative of G_{mix}, we have to take into account that ϖ contains N_ν according to Eq. (S14). Thus

$$\begin{aligned}
\frac{\partial G_{\mathrm{mix}}}{\partial N_\nu} = -\beta^{-1} \Bigg[& N_{\mathrm{W}} \frac{N_{\mathrm{W}} \omega_{\mathrm{W}}}{\varpi} \frac{\omega_\nu}{N_{\mathrm{W}} \omega_{\mathrm{W}}} + N_{\mathrm{P}} \frac{N_{\mathrm{P}} \omega_{\mathrm{P}}}{\varpi} \frac{\omega_\nu}{N_{\mathrm{P}} \omega_{\mathrm{P}}} + \sum_{\substack{\nu' \\ \nu' \neq \nu}} N_{\nu'} \frac{N_{\nu'} \omega_{\nu'}}{\varpi} \frac{\omega_\nu}{N_{\nu'} \omega_{\nu'}} \\
& + \ln\left\{\frac{\varpi}{N_\nu \omega_\nu}\right\} + N_\nu \frac{N_\nu \omega_\nu}{\varpi} \left(\frac{\omega_\nu}{N_\nu \omega_\nu} - \frac{\varpi}{N_\nu^2 \omega_\nu} \right) + \sum_\alpha N_\alpha \frac{N_\alpha \omega_\alpha}{\varpi} \frac{\omega_\nu}{N_\alpha \omega_\alpha} \Bigg] \\
= -\beta^{-1} \Bigg[& \ln\left\{\frac{\varpi}{N_\nu \omega_\nu}\right\} + \frac{\omega_\nu}{\varpi} \left\{ N_{\mathrm{W}} + N_{\mathrm{P}} + \sum_{\substack{\nu' \\ \nu' \neq \nu}} N_{\nu'} + \sum_\alpha N_\alpha + N_\nu \left(1 - \frac{\varpi}{N_\nu \omega_\nu} \right) \right\} \Bigg] \\
= -\beta^{-1} & \left[\bar{N} \frac{\omega_\nu}{\varpi} - \ln\left\{\frac{N_\nu \omega_\nu}{\varpi}\right\} - 1 \right] = \beta^{-1} \left[1 + \ln\left\{\frac{N_\nu \omega_\nu}{\varpi}\right\} - \bar{N} \frac{\omega_\nu}{\varpi} \right]
\end{aligned} \tag{S19}$$

For the derivative of G_{int}, we have to take into account that $\bar{N}$ contains N_ν according to Eq. (9) of the main text:

$$\frac{\partial G_{\mathrm{int}}}{\partial N_\nu} = \beta^{-1} \left[J_\nu \frac{N_{\mathrm{P}}}{\bar{N}} - \sum_{\nu'} J_{\nu'} \frac{N_{\mathrm{P}} N_{\nu'}}{\bar{N}^2} \right] \tag{S20}$$

In the limit of diluted detergent, we can make the approximation $\bar{N} \approx N_{\mathrm{tot}}$, which leads to

$$\frac{\partial G_{\mathrm{int}}}{\partial N_\nu} = \beta^{-1} \left[J_\nu X_{\mathrm{P}} - \sum_{\nu'} J_{\nu'} X_{\mathrm{P}} \frac{X_{\nu'}}{\nu'} \right] \tag{S21}$$

where one has to recall the definition of X_ν in Eq. (7) of the main text. Based on Eq. (12) of the main text and using Eqs. (S18), (S19), and (S21), we can compute

$$-\beta\nu(\mu_\nu^{\mathrm{o}} - \mu_1^{\mathrm{o}}) = \ln\left\{\frac{N_\nu \omega_\nu \varpi^\nu}{N_1^\nu \omega_1^\nu \varpi}\right\} + 1 - \nu + \bar{N} \frac{\nu\omega_1 - \omega_\nu}{\varpi} + X_{\mathrm{P}}(J_\nu - \nu J_1) + (\nu - 1) \sum_{\nu'} J_{\nu'} X_{\mathrm{P}} \frac{X_{\nu'}}{\nu'} \tag{S22}$$

A further simplification can be achieved, if the assumption is made that the free volumes ω_i are proportional to the molar volumes v_i with a species-independent proportionality constant. Then, the volume fraction Y_i of species i (with mole number n_i and molarity c_i) becomes

$$Y_i = \frac{N_i \omega_i}{\varpi} = \frac{N_i \mathrm{v}_i}{V} = \frac{n_i v_i}{V} = c_i v_i = c_{\mathrm{tot}} v_i X_i \tag{S23}$$

with the consequence that

$$\frac{\omega_i}{\varpi} = \frac{\mathrm{v}_i}{V} = \frac{v_i}{N_\mathrm{A} V} \tag{S24}$$

where N_A is Avogadro´s number, so that

$$\bar{N}\frac{\omega_i}{\varpi} = \frac{\bar{N} v_i}{N_\mathrm{A} V} \approx c_{\mathrm{tot}} v_i \tag{S25}$$

Here, we again assumed $\bar{N} \approx N_{\mathrm{tot}}$ and $c_{\mathrm{tot}} \approx \bar{N}/(N_\mathrm{A} V)$. Then

$$-\beta\nu(\mu_\nu^\mathrm{o} - \mu_1^\mathrm{o}) = \ln\left\{\frac{X_\nu}{\nu X_1^\nu}\right\} + \ln\left\{\frac{c_{\mathrm{tot}} v_\nu}{(c_{\mathrm{tot}} v_1)^\nu}\right\} + 1 - \nu + c_{\mathrm{tot}}(\nu v_1 - v_\nu)$$

$$+X_\mathrm{P}\left[J_\nu - \nu J_1 + (\nu - 1)\sum_{\nu'} J_{\nu'} \frac{X_{\nu'}}{\nu'}\right] \tag{S26}$$

In Eq. (S26), the terms have the following physicochemical meaning:

(a) Equilibrium constant

The first term on the right hand side in the first line is simply the logarithm of the equilibrium constant for the formation of a micelle with aggregation number ν from ν detergent monomers as formulated for a specific aggregation number m in Eq. (53) of the main text.

(b) Molecular volume effects

The remaining terms on the right hand side in the first line originate from the consideration of molecular volumes in the entropy of mixing. These terms vanish in an ideal-mixing model as the one used in the main text based on Eq. (8). Of particular note is the last term that depends on the molar volume difference between a micelle with aggregation number ν (v_ν) and ν detergent monomers (νv_1). This difference can be argued to be small, so that this term could be neglected. However, the other term containing c_{tot} and the term $1 - \nu$ would not vanish and cause a difference between equilibrium constants formulated either with volume fractions or mole fractions. Volume fractions are a better choice in self-aggregation problems, but mole fractions are easier to fix in experiments.

(c) Detergent-PEG interaction

The terms in the second line that depend on X_P describe the interaction of PEG with the detergent involving all possible aggregate sizes of the latter.

Solving Eq. (S26) for X_ν yields the micellar size distribution. It can be seen that this size distribution – and hence the average aggregation number – depends on the molecular volume effects and the detergent-PEG interaction. However, determining the coupling constants J_ν and J_1 is challenging, so that a faithful prediction of the effect of PEG on the aggregation number is difficult.

In a mass-action model, only one type of micelle with a fixed aggregation number $\nu = m$ exists besides detergent monomers. Note that within such a model, no change of the aggregation number by PEG can be modeled. However, Eq. (S26) becomes:

$$-\beta m(\mu_m^{\text{o}} - \mu_1^{\text{o}}) = \ln\left\{\frac{X_m}{mX_1^m}\right\} + \ln\left\{\frac{c_{\text{tot}}v_m}{(c_{\text{tot}}v_1)^m}\right\} + 1 - m + c_{\text{tot}}(mv_1 - v_m)$$

$$+X_{\text{P}}\left[J_m - mJ_1 + (m-1)\left(J_1X_1 + J_m\frac{X_m}{m}\right)\right] \quad \text{(S27)}$$

Besides $y = X_1$, $z = X_m$, and $g_{\text{mic}} = \beta(\mu_m^{\text{o}} - \mu_1^{\text{o}})$ introduced in the main text, we define the following quantities: (i) the volume difference between a micelle with aggregation number m and m detergent monomers per detergent molecule in the micelle

$$\Delta v_m = \frac{1}{m}(v_m - mv_1) = \frac{v_m}{m} - v_1 \quad \text{(S28)}$$

(corresponding to $-\xi$ used by Bothe et al.[2]) and (ii) the difference in interaction of PEG with a micelle with aggregation number m and with m detergent monomers per detergent molecule in the micelle

$$\Delta j_m = \frac{1}{m}(J_m - mJ_1) = \frac{J_m}{m} - J_1 \quad \text{(S29)}$$

(denoted ζ by Bothe et al.[2]). With these settings, Eq. (S27) becomes

$$-mg_{\text{mic}} = \ln\left\{\frac{z}{my^m}\right\} + \ln\left\{\frac{c_{\text{tot}}v_m}{(c_{\text{tot}}v_1)^m}\right\} + 1 - m(1 + c_{\text{tot}}\Delta v_m)$$

$$+mX_{\text{P}}\Delta j_m + (m-1)X_{\text{P}}(J_1x + \Delta j_m z)$$

$$= \ln\left\{\frac{z}{my^m}\right\} + \ln\left\{\frac{c_{\text{tot}}v_m}{(c_{\text{tot}}v_1)^m}\right\} + 1 - m(1 + c_{\text{tot}}\Delta v_m)$$

$$+X_{\text{P}}\Delta j_m[m - (m-1)y] + (m-1)X_{\text{P}}\frac{J_m}{m}x \quad \text{(S30)}$$

For the sake of clarity, let us assume that Δv_m is small and can be disregarded. Also, we introduce further abbreviations: The factor containing the detergent monomer concentration y accompanying X_{P} will be denoted by

$$\bar{\xi}(y) = \Delta j_m[m - (m-1)y] \quad \text{(S31)}$$

It reduces to $\xi(y)$ in Eq. (19) of the main text, if we neglect the PEG-micelle interaction represented by the coupling constant J_m and write J for J_1. The terms containing the total detergent concentration x will be written as follows:

$$\eta(x) = (m-1)x\frac{J_m}{m} \quad \text{(S32)}$$

Finally, we introduce

$$\Psi = \ln\left\{\frac{c_{\text{tot}}v_m}{(c_{\text{tot}}v_1)^m}\right\} = \ln\left\{\frac{c_{\text{tot}}v_m m^m}{(c_{\text{tot}}v_m)^m}\right\} = (1-m)\ln\{c_{\text{tot}}v_m\} + m\ln m \tag{S33}$$

where we used $v_m \approx m v_1$, and

$$\psi = \frac{1}{m}(\Psi + 1 - m) = \frac{\Psi}{m} + \frac{1}{m} - 1 \tag{S34}$$

as done by Bothe et al.[2] With these abbreviations, we can write down an extended version of Eq. (21) of the main text:

$$x = y + m y^m \exp\{-m[g_{\text{mic}} + \psi] - X_{\text{P}}[\bar{\xi}(y) + \eta(x)]\} \tag{S35}$$

It can be seen that now the exponent is a function of x and y, which considerably complicates the further analysis based on Eq. (22) compared to the simple model in the main text.

In the derivation of Eq. (36) of the main text, the assumption was made that the ratio y/x at the CMC given in Eq. (35) is not significantly changed by the PEG-detergent interaction in the limit of large micelles, so that Eq. (35) could be plugged into Eq. (21) to proceed. This procedure was justified for the simple model, in which only PEG-monomer interaction is taken into account, above in "Derivation and Discussion of Eq. (36)". For the extended model, it remains to be shown that Eq. (35) is still a good approximation in the limit $m \to \infty$. Assuming, though, that it is a good approximation, we can plug Eq. (35) into Eq. (S35) to obtain:

$$\ln X_{\text{CMC}} = \frac{m}{m-1}[g_{\text{mic}} + \psi] - \frac{1}{m-1}\ln\left\{\frac{m(2m^2-m)^m}{(m-2)(2m^2-2)^{m-1}}\right\}$$

$$+X_{\text{P}}\left[\Delta j_m\left[\frac{m}{m-1} - X_{\text{CMC}}\right] + X_{\text{CMC}}\frac{J_m}{m}\right] \tag{S36}$$

In the limit of large m, $m/(m-1) \to 1$, so that

$$\ln X_{\text{CMC}} = g_{\text{mic}} + \psi - \frac{1}{m-1}\ln\left\{\frac{m(2m^2-m)^m}{(m-2)(2m^2-2)^{m-1}}\right\} + X_{\text{P}}\left[\Delta j_m(1 - X_{\text{CMC}}) + X_{\text{CMC}}\frac{J_m}{m}\right]$$

$$= g_{\text{mic}} + \psi - \frac{1}{m-1}\ln\left\{\frac{m(2m^2-m)^m}{(m-2)(2m^2-2)^{m-1}}\right\}$$

$$+X_{\text{P}}\left[\frac{J_m}{m} - J_1 - \frac{J_m}{m}X_{\text{CMC}} + J_1 X_{\text{CMC}} + X_{\text{CMC}}\frac{J_m}{m}\right]$$

$$= g_{\text{mic}} + \psi - \frac{1}{m-1}\ln\left\{\frac{m(2m^2-m)^m}{(m-2)(2m^2-2)^{m-1}}\right\} + X_{\text{P}}\left[\frac{J_m}{m} + J_1(X_{\text{CMC}} - 1)\right] \tag{S37}$$

If we denote by $X_{\text{CMC}}(0)$ and $\psi(0)$ the CMC and the quantity ψ, respectively, at zero PEG concentration, we obtain:

$$\ln\frac{X_{\text{CMC}}}{X_{\text{CMC}}(0)} = \psi - \psi(0) + X_{\text{P}}\left[\frac{J_m}{m} + J_1(X_{\text{CMC}} - 1)\right] \tag{S38}$$

The simple form

$$\ln \frac{X_{\mathrm{CMC}}}{X_{\mathrm{CMC}}(0)} = \kappa X_{\mathrm{P}} \tag{S39}$$

corresponding to Eq. (38) of the main text is obtained, if we neglect excluded-volume effects (i. e., set $\psi = \psi(0)$) and define:

$$\kappa = \frac{J_m}{m} + J_1(X_{\mathrm{CMC}} - 1) \tag{S40}$$

Taking into account that $X_{\mathrm{CMC}} \ll 1$, this can be further simplified to

$$\kappa = \frac{J_m}{m} - J_1 \tag{S41}$$

Thus, κ is the difference of the coupling constant specifying detergent-PEG interaction between a detergent molecule in the micelles (or that of a micelle per detergent molecule in the micelle) and a detergent monomer in the aqueous environment.
